# Inhibiting spinal cord-specific hsp90 isoforms reveals a novel strategy to improve the therapeutic index of opioid treatment

**DOI:** 10.1038/s41598-024-65637-6

**Published:** 2024-06-26

**Authors:** David I. Duron, Parthasaradhireddy Tanguturi, Christopher S. Campbell, Kerry Chou, Paul Bejarano, Katherin A. Gabriel, Jessica L. Bowden, Sanket Mishra, Christopher Brackett, Deborah Barlow, Karen L. Houseknecht, Brian S. J. Blagg, John M. Streicher

**Affiliations:** 1 Department of Pharmacology, College of Medicine, University of Arizona, Box 245050, LSN563, 1501 N. Campbell Ave., Tucson, AZ 85724 USA; 2 Department of Chemistry and Biochemistry, College of Science, University of Notre Dame, Notre Dame, IN USA; 3 Department of Biomedical Sciences, College of Osteopathic Medicine, University of New England, Biddeford, ME USA; 4 Comprehensive Center for Pain and Addiction, University of Arizona, Tucson, AZ USA

**Keywords:** Heat shock protein 90, Opioid, Anti-nociception, Reward, Constipation, Tolerance, Therapeutic index, Isoforms, Sensory processing, Neuroscience, Molecular neuroscience

## Abstract

Opioids are the gold standard for the treatment of chronic pain but are limited by adverse side effects. In our earlier work, we showed that Heat shock protein 90 (Hsp90) has a crucial role in regulating opioid signaling in spinal cord; Hsp90 inhibition in spinal cord enhances opioid anti-nociception. Building on these findings, we injected the non-selective Hsp90 inhibitor KU-32 by the intrathecal route into male and female CD-1 mice, showing that morphine anti-nociceptive potency was boosted by 1.9–3.5-fold in acute and chronic pain models. At the same time, tolerance was reduced from 21-fold to 2.9 fold and established tolerance was rescued, while the potency of constipation and reward was unchanged. These results demonstrate that spinal Hsp90 inhibition can improve the therapeutic index of morphine. However, we also found that systemic non-selective Hsp90 inhibition blocked opioid pain relief. To avoid this effect, we used selective small molecule inhibitors and CRISPR gene editing to identify 3 Hsp90 isoforms active in spinal cord (Hsp90α, Hsp90β, and Grp94) while only Hsp90α was active in brain. We thus hypothesized that a systemically delivered selective inhibitor to Hsp90β or Grp94 could selectively inhibit spinal cord Hsp90 activity, resulting in enhanced opioid therapy. We tested this hypothesis using intravenous delivery of KUNB106 (Hsp90β) and KUNG65 (Grp94), showing that both drugs enhanced morphine anti-nociceptive potency while rescuing tolerance. Together, these results suggest that selective inhibition of spinal cord Hsp90 isoforms is a novel, translationally feasible strategy to improve the therapeutic index of opioids.

## Introduction

Opioid drugs like morphine are the gold standard for the treatment of moderate to severe chronic pain, but are limited by serious side effects, including tolerance, constipation, and reward/addiction liability^1,2^. These limitations have spurred the search for alternate approaches to improve opioids, such as multifunctional ligands, or opioid agonists biased against the recruitment of βarrestin2 (recently reviewed in Ref.^3^). While none of these approaches has solved the opioid issue, they have revealed how manipulating the signal transduction cascades of the opioid receptors holds great promise in improving opioid outcomes (e.g., arrestin bias).

To this end, we’ve engaged in a long-term effort to investigate the role of Heat shock protein 90 (Hsp90) in regulating opioid signaling and anti-nociception. Hsp90 is a ubiquitous and highly expressed chaperone protein with a variety of roles, including nascent protein maturation, signaling kinase activation, and scaffolding signaling complex formation (reviewed in Refs.^4,5^). Hsp90 has mostly been studied in the context of cancer. A few papers have shown that Hsp90 promotes inflammation during inflammatory and neuropathic pain^6–8^, and another two papers have shown that Hsp90 could promote opioid dependence and withdrawal^9,10^. More recently, another paper has linked the Hsp90β isoform to opioid receptor signaling^11^. However, in general, the role of Hsp90 in the pain and opioid systems is mostly unstudied (reviewed in Ref.^12^).

In our work, we’ve uncovered a role for Hsp90 in opioid signaling and anti-nociception that differs between the brain and spinal cord. In brain, Hsp90 promotes ERK MAPK signaling via the Hsp90α isoform and the co-chaperones Cdc37 and p23; Hsp90 inhibitor treatment in the brain thus reduces ERK MAPK signaling and opioid anti-nociception^13–15^. In contrast, Hsp90 represses opioid anti-nociception and signaling in the spinal cord, so that Hsp90 inhibitor treatment in the spinal cord promotes an ERK-RSK signaling cascade that results in enhanced opioid anti-nociception^16^. Notably, in this study, we only tested one opioid dose, and did not test any opioid side effects, thus we could not determine if the therapeutic index of opioids had been improved.

This enhanced anti-nociception led us to hypothesize that spinal Hsp90 inhibitor treatment could be used to improve the therapeutic index of opioids and enable a dose-reduction strategy. This is because many side effects are regulated outside the spinal cord, and would presumably not be impacted by spinal inhibition (e.g. reward in ventral tegmental area and striatum^17^, constipation in the gut^18^). If anti-nociception were improved but side effects were either improved or not altered, then a lower dose of opioid could be given in combination with Hsp90 inhibitor treatment. This would hypothetically result in improved or maintained anti-nociception with decreased side effects (see Ref.^19^ for a parallel approach). A novel dose-reduction strategy of this kind could be used to improve opioid therapy in chronic pain patients, decreasing the impact of the negative side effects of opioid therapy.

## Materials and methods

### Drugs and CRISPR constructs

KU-32, KUNA115, KUNB106, and KUNG65 were synthesized, purified, and characterized as in our previously published work (KU-32 is Compound A4 in Ref.^20^; KUNA115 in Ref.^21^; KUNB106 in Ref.^22^; KUNG65 in Ref.^23^). Purity was confirmed by HPLC (> 95%) and identity confirmed by HRMS and NMR. Inhibitors were stored under desiccation at –20 °C, and stock solutions were prepared in DMSO, and also stored at –20 °C. Naloxone (#AAJ60013MC) was obtained from Fisher Scientific (Waltham, MA). A matched Vehicle control injection was included in every experiment; 0.02% DMSO in sterile USP water for the 0.01 nmol intrathecal injections and 10% DMSO, 10% Tween80, and 80% sterile USP saline for 1–10 mg/kg intravenous injections. Morphine sulfate pentahydrate was obtained from the NIDA Drug Supply Program, stored at room temperature, and working solutions were made fresh prior to every experiment in sterile USP saline. USP saline-injected controls were used for the reward and constipation assays below, as well as for the naloxone injections.

All-in-one CRISPR DNA constructs expressing Cas9 and a gRNA targeting Hsp90α (MCP229411-CG01-3-B), Hsp90β (MCP227368-CG12-3-B), and Grp94 (MCP230394-CG12-3-B) were obtained from Genecopoeia (Rockville, MD). The DNA was amplified using standard molecular biology approaches, and complexed with TurboFect in vivo transfection reagent (Thermo Fisher, Waltham, MA) as described in our previous work^14^ and by the manufacturer’s protocol. The complexed DNA was injected into the mice by the intrathecal route (2 μg DNA in 5 μL) daily from days 1–3, with behavioral testing performed on day 10.

### Mice

Male and female CD-1 mice in age-matched cohorts from 5 to 8 weeks of age were used for all behavioral experiments and were obtained from Charles River Laboratories (Wilmington, MA). CD-1 (a.k.a. ICR) mice are commonly used in opioid research as a line with a strong response to opioid drugs (*e.g.* Ref.^24^, and our own previous Hsp90 research^13–16^). Mice were recovered for a minimum of 5 days after shipment before being used in experiments. Mice were housed no more than 5 mice per cage and kept in an AAALAC-accredited vivarium at the University of Arizona under temperature control and 12-h light/dark cycles. All mice were provided with standard lab chow and water available ad libitum. The animals were monitored daily, including after surgical procedures, by trained veterinary staff. All experiments performed were in accordance with IACUC-approved protocols at the University of Arizona and by the guidelines of the NIH Guide for the Care and Use of Laboratory Animals. We also adhered to the guidelines of ARRIVE; no adverse events were noted for any of the animals.

### Behavioral experiments

All animals were randomized to treatment groups by random assignment of mice in one cohort to cages, followed by random block assignment of cages to treatment group. Group sizes were based on previous published work from our lab using these assays^14,16,25,26^. The mice were not habituated to handling. Prior to any behavioral experiment or testing, animals were brought to the testing room in their home cages for at least 1 h for acclimation. Testing always occurred within the same approximate time of day between experiments during the animal light (inactive) cycle, and environmental factors (noise, personnel, and scents) were minimized. All testing apparatus (cylinders, grid boxes, etc.) were cleaned between uses using 70% ethanol and allowed to dry. The experimenter was blinded to treatment group by another laboratory member delivering coded drug vials, which were then decoded after collection of all data. Naïve mice were used for every experiment, including each dose. For morphine dosing in each assay, the first group was started with a moderate dose, usually 3.2 mg/kg. Based on this response, the dosing in each assay would be adjusted up or down by half- or quarter-log steps to maintain at least 3 responses within the linear range of the assay between baseline and threshold.

### Paw incision and mechanical allodynia

Mechanical thresholds were determined prior to surgery using calibrated von Frey filaments (Ugo Basile, Varese, Italy) with the up-down method and four measurements after the first response per mouse as in Ref.^27^ and our previously published work (e.g. Ref.^28^). The mice were housed in a homemade apparatus with Plexiglas walls and ceiling and a wire mesh floor (3-inch wide 4-inch long 3-inch high with 0.25-inch wire mesh). The surgery was then performed by anesthesia with ~ 2% isoflurane in standard air, preparation of the left plantar hind paw with iodine and 70% ethanol, and a 5-mm incision made through the skin and fascia with a no. 11 scalpel. The muscle was elevated with curved forceps leaving the origin and insertion intact, and the muscle was split lengthwise using the scalpel. The wound was then closed with 5–0 polyglycolic acid sutures. Mice were then injected with inhibitor or Vehicle control and left to recover for 24 h. Our intrathecal (i.t.) injection protocol is reported in Refs.^13,29^; briefly, the injection was made in awake and restrained animals with a 10 μL Hamilton syringe and 30 g needle (5–7 μL volume) between the L5-L6 vertebrae at a 45° angle, with placement validated by tail twitch. Since the injection was made through skin with no incision or other surgical intervention, repeated injection protocols were performed the same way. The next day, the mechanical threshold was again determined as described above. Mice were then injected with 0.32–5.6 mg/kg morphine by the subcutaneous (s.c.) route, and mechanical thresholds were determined over a 3-h time course. No animals were excluded from these studies.

### Tail-flick assay

Pre-injection tail-flick baselines were determined in a 52 °C tail-flick assay with a 10-s cutoff time (method also reported in Ref.^13^). The mice were then injected with inhibitor or Vehicle control with a 24-h treatment time. 24-h post-injection baselines were determined. The mice were then injected s.c. with 1–10 mg/kg of morphine, and tail flick latencies were determined over a 2-h time course. For tolerance studies, baseline tail flick latencies were taken, and mice were then injected with inhibitor or Vehicle control with a 24-h treatment time. 24 h later mice were baselined again and then injected with 10 mg/kg s.c. morphine with one tail flick latency measured at 30 min post morphine. Mice were injected again with inhibitor or Vehicle and the process was repeated for an additional 7 days with twice daily morphine injection, and tail flick response measured after the morning injection. No animals were excluded from these studies.

### HIV peripheral neuropathy

Mechanical threshold baselines were measured prior to any treatment on the left hind paw using von Frey filaments. HIV peripheral neuropathy was induced by intrathecal injection of gp120 IIIb protein (SPEED BioSystems, Gaithersburg, MD, Cat# YCP1549, 15 ng/μl in 0.1 M PBS and 0.1% BSA, 7-μl volume) using our previously established protocol^13^ on days 1, 3, and 5. On day 20 a second mechanical threshold baseline was measured on the left hind paw using von Frey filaments and then KU-32 or Vehicle was injected i.t with a 24-h treatment time. A third mechanical threshold was then measured on day 21 and morphine (0.32–10 mg/kg s.c.) was then injected, and mechanical thresholds were measured over a time course on the left hind paw. No animals were excluded from these studies.

### Conditioned place preference

Conditioned place preference training, baseline runs, and post-training runs were all performed in Spatial Place Preference LE 896/898 rigs (Harvard Apparatus, Holliston, MA). Rigs were designed to consist of two chambers with one connecting chamber. Of the two conditioned chambers, one consisted of black and grey dotted walls with a textured floor. The other chamber consisted of black and grey striped walls with smooth floor. Chamber floors connected to a pressure sensor which transferred ongoing data to a computer running PPC WIN 2.0 software (Harvard Apparatus). Prior to preference training baselines were taken on day 0. Mice were placed in CPP chambers and allowed to roam freely for 15 min at ~ 7am. Chambers were cleaned thoroughly with VersaClean and allowed to dry in-between mice. Mice were then injected with i.t. KU-32 or Vehicle with a 24-h treatment time. On day 1 mice were injected with i.t. KU-32 or Vehicle again and allowed to recover for 30 min. Mice were then injected s.c. with saline or morphine (3.2, 5.6, or 10 mg/kg) at ~ 7am and placed in either stripe or dotted chambers. Half of each group paired morphine with the striped chamber and the other half to the dotted chamber in an unbiased design. At ~ 12 pm mice were then given a second injection of either saline or morphine which was paired to the opposite chamber. This training process was repeated for 4 days total with morning and noon pairings alternating each day. On day 5 mice were placed in CPP chambers and allowed to roam freely for 15 min at ~ 7am. Raw data in the form of seconds and percentage spent in each chamber was exported from PPC WIN 2.0 as an excel file and transferred to GraphPad Prism 9.3 (San Diego, CA) for further analysis.

### Opioid induced constipation

Prior to the experiment mice were injected with either KU-32 or Vehicle i.t. and allowed to recover for 24 h. Morphine (1, 3.2, or 10 mg/kg s.c.) or saline was injected and followed by a 6-h fecal production time course. During this time course the mice were housed in the von Frey boxes used to collect the paw incision and HIV neuropathy data above, which have a grate above a collection plate. The feces were counted and weighed in 1-h bins and used to construct a cumulative plot. Morphine-treated groups were normalized to saline groups and represented as a percentage at each timepoint.

### Respiratory depression

Respiratory activity and subsequent morphine-induced depression was measured using whole body plethysmography in awake and freely moving mice using chambers from Data Sciences International (St. Paul, MN). Chambers were maintained at room temperature, with composition of the atmosphere set by mass flow controllers. Mice were injected with inhibitors or Vehicle control, followed by 24 h treatment time, as for the above assays. The mice were then placed in the chambers for a 30-min acclimation and baseline period, with respiratory measurements recorded for the last 7 min of the period. All mice were then treated with 7.5 mg/kg morphine i.v., immediately placed back in the chambers, and respiratory activity recorded for an additional hour.

### In vitro *ADME assays*

#### LogD determination

Distribution coefficient (LogD) was determined by the method of Wilson et al.^30^. Briefly, 5 µL volumes of test compounds diluted in DMSO were added to a mixture of equal volumes of 50 mM phosphate buffer and 1-octanol. The compounds were assayed using a 50 nM concentration in order to limit compound precipitation and to ensure that the assay values were maintained within the dynamic range of the LC–MS/MS instrumentation. Samples were vortex mixed at 800 rpm for 24 h. Subsequent to centrifugation at 14,000 rpm for 30 min, 1 µL of each layer was analyzed by LC–MS/MS. LogD was calculated using the peak areas obtained from each layer.

#### Aqueous solubility

Aqueous solubility was determined using a miniaturized shake flask approach, under conditions of pH 6.8 and analyte concentration of 1.0 mM by the method of Zhou et al.^31^. Aqueous solutions of analyte were incubated at room temperature in the chamber of a Whatman (Piscataway, NJ) Mini-UniPrep syringeless filter for 24 h while shaking gently (600 rpm). Subsequent to incubation, filter plungers were pushed down to the bottom of the syringeless filter chamber assemblies, allowing filtrate to enter the plunger compartment. Following an additional 30 min incubation at room temperature, filtrates were diluted with 50:50 acetonitrile/water + 0.1% formic acid and analyzed by LC − MS/MS. Analyte concentrations were determined by the interpolation of peak area ratio from a calibration curve formed by matrix spiked with authentic reference material.

#### In vitro mouse liver microsomal stability

Metabolic stability of lead compounds was assessed in vitro by the method of Di et al.^32^. Briefly, mouse liver microsomes (Corning Life Sciences, Woburn, MA) were isolated from CD-1 mice (male mice, 8 − 10 weeks of age). Assays were conducted using 0.123 mg/mL protein concentration (total protein concentration in the microsomal solution) and 1.0 μM drug concentration under incubation conditions of 37 °C. Metabolic stability was determined following 0, 5, 15, 30, and 60 min of incubation time. The samples were analyzed by reversed phase LC using a triple quadrupole mass spectrometer. Compound specific transitions of parent ion to product ion were monitored and percent remaining calculated based on peak area of 5 − 60 min time points (relative to time zero). Half-life calculations were determined using the formula t½ =  − ln(2)/k, where k (min^−1^) is the turnover rate constant (the slope) estimated from a log − linear regression of the percentage compound remaining versus time.

#### In vitro human liver microsomal stability

Metabolic stability of the compounds in human liver microsomes was determined by the method described above using pooled human liver microsome preparations from 20 male donors (Corning Life Sciences, Woburn, MA). Assays were conducted as described above and the in vitro half-life of the compounds was calculated.

#### LC − MS/MS analysis

LC − MS/MS analysis was conducted using an Agilent (Santa Clara, CA) 6460 triple quadrupole mass spectrometer coupled with an Agilent liquid chromatography (LC) system. The LC system consists of a binary pump, degasser, column heater, and autosampler. Chromatographic separation was performed on a Waters Atlantis T3 3 µm 3.0 × 50 mm analytical column using a ballistic gradient of mobile phase consisting of 0.1% formic acid in water (A) and 0.1% formic acid in acetonitrile (B) at a flow rate of 0.75 mL/min. The mobile phase was heated to a temperature of 45 °C.

### In vivo *pharmacokinetic study*

Mice were dosed with 1–10 mg/kg KUNG65 by the intravenous route, with groups over a 2 h time course. The mice from each time point were sacrificed, and whole blood collected into EDTA tubes from a cardiac puncture. This was followed by saline perfusion to clear the vasculature, and the spinal cords were dissected and snap frozen in liquid nitrogen. The whole blood was separated and the plasma stored in EDTA tubes; all samples were stored at − 80 °C prior to analysis. Compound exposure in plasma subsequent to in vivo dosing was determined by precipitation of protein with acetonitrile. Following centrifugation, the supernatant was injected for LC–MS/MS analysis, and plasma concentration determined via interpolation of peak areas from a standard curve prepared in plasma.

### Competition radioligand binding

KUNB106 binding to the opioid receptors was performed substantially as in our previous work^33–37^. Human mu (#ES-542-C), delta (#RBHODM-K), and kappa (#ES-541-C) opioid-expressing CHO cell lines from PerkinElmer (Waltham, MA) were used. The cells were grown in in 1:1 DMEM/F12 medium with 10% heat-inactivated fetal bovine serum, 1X penicillin–streptomycin supplement, and 500 μg/mL G418 selection antibiotic. Cell pellets for experiments were collected using 5 mM EDTA in PBS and membrane protein extracted as described in our cited work. For the binding, 25–30 μg of membrane protein was combined with a fixed concentration (0.57–5.18 nM) of ^3^H-diprenorphine (PerkinElmer) and concentration curves of KUNB106 or positive control (naloxone for mu and delta, U50,488 for kappa) in a 200 μL volume. The reactions were incubated for 1 h at room temp, then collected onto 96 well format GF/B filter plates using a Brandel Cell Harvester (Gaithersburg, MD). The radioactivity was read using a 96 well format MicroBeta2 scintillation counter (PerkinElmer). The resulting data were normalized to binding in the presence of Vehicle (100%) or 10 μM naloxone/U50,488 (0%), and used to calculate the K_I_ based on the previously established K_D_ of ^3^H-diprenorphine in each cell line using GraphPad Prism 9.3 using a 1-site fit model.

### Statistical analysis

All data were reported as the mean ± SEM and normalized where appropriate as described above. The behavioral data from each individual dose were reported raw without maximum possible effect (MPE) or other normalization. Data for dose/response curves (except CPP) were normalized to %MPE using peak effect, except for constipation, which used Area Under the Curve [MPE = (Response-Baseline)/(Threshold – Baseline) * 100]. The CPP dose/response curve was reported as the % Difference Score [Diff Score = % in Paired—% in Unpaired]. Technical replicates and further details are described in the Figure Legends. Potency (A_50_) values were calculated by linear regression using our previously reported method^13^, with further details in the Figure Legends, and were reported with 95% confidence intervals. Statistical comparisons of individual dose/response curve time courses were performed using Repeated Measures 2-Way ANOVA with Sidak’s (tail flick, paw incision, HIV neuropathy, tolerance rescue, constipation, respiratory depression) or Tukey’s (tolerance, CPP) post hoc tests. The Geisser-Greenhouse correction was used to account for a potential lack of sphericity of the data, permitting valid Repeated Measures ANOVA. ANOVA post hoc tests were only performed when ANOVA F values indicated a significant difference, and there was homogeneity of variance (permitting parametric analysis). In all cases, significance was defined as p < 0.05. The group sizes reported represent independent individual mice tested in each assay. All graphing and statistical analyses were performed using GraphPad Prism 9.3. Approximately equal numbers of male and female mice were used for each experiment. Comparison by 2 Way ANOVA using sex as a variable revealed no sex differences in this study, so all male and female mice were combined.

## Results

Each experiment included approximately equal numbers of male and female mice. Comparison by 2-Way ANOVA using sex as a variable revealed no sex differences in this study, so all male and female mice were combined.

### Spinal Hsp90 inhibition enhances morphine anti-nociception in multiple pain models

**Figure 1:**
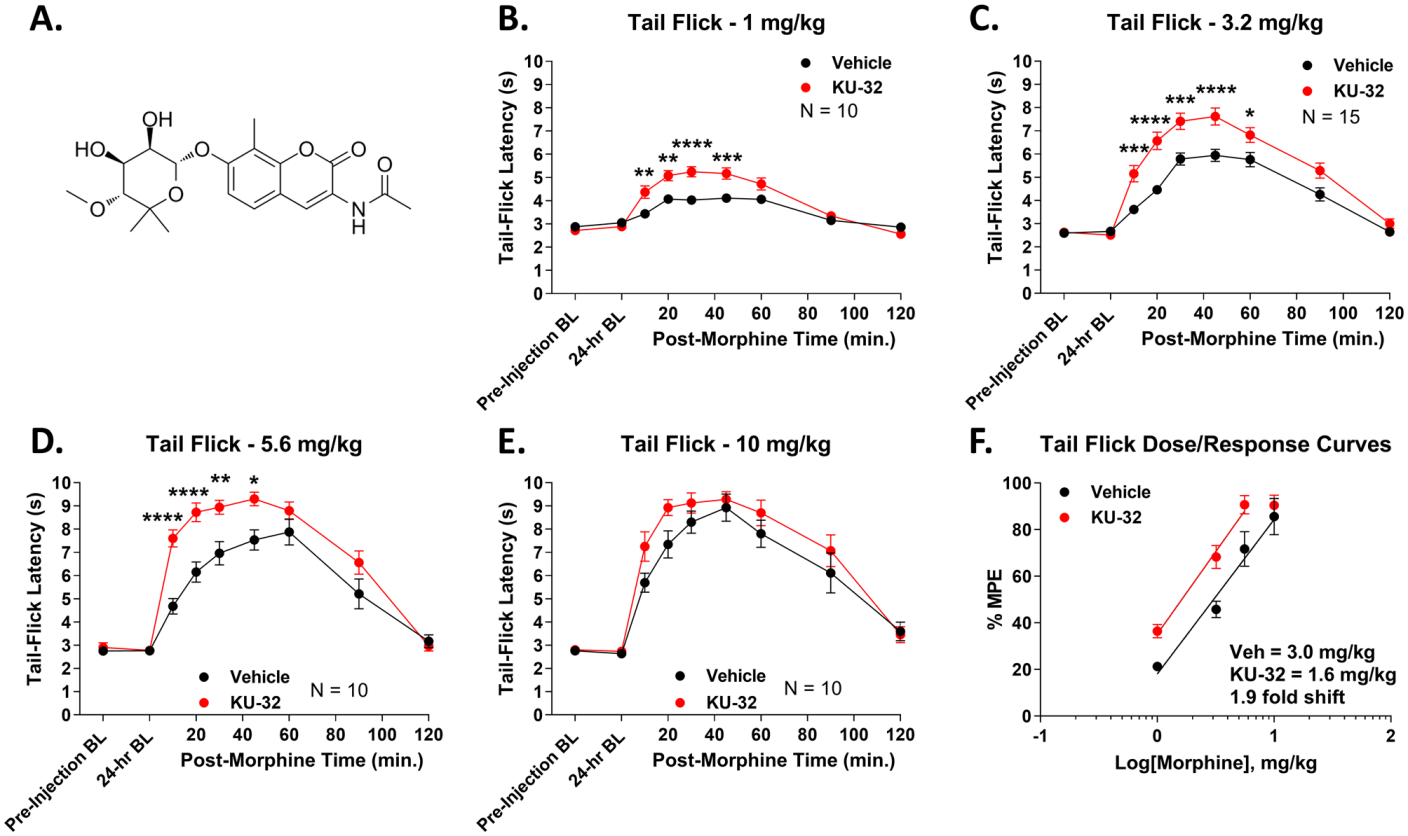
Spinal Hsp90 inhibition increases morphine potency in acute heat-induced tail flick. Male and female CD-1 mice were injected i.t. with 0.01 nmol KU-32 (**A**) or Vehicle control, 24 h, then 1–10 mg/kg morphine, s.c.. Males and females did not differ by 2 Way ANOVA (p > 0.05), so all male and female data were combined. Tail flick responses were recorded at 52 °C with a 10 s cutoff. Data were reported as the mean ± SEM, with sample sizes of mice/group noted in each graph. 2–3 technical replicates were performed per dose. *, **, ***, **** = p < 0.05, 0.01, 0.001, 0.0001 vs. same time point Vehicle group by RM 2 Way ANOVA with Sidak’s post hoc test. (**B**–**E**) Individual dose curves shown as noted. The data were not normalized. (**F**) Dose/response analysis was performed for individual curves, normalized as %MPE (baseline vs. 10 s cutoff), with linear regression for A_50_ calculation (see “Methods”). A_50_: Vehicle = 3.0 (2.5–3.7) mg/kg; KU-32 = 1.6 (1.3–2.0) mg/kg; 1.9-fold increase in potency.

In our earlier work, we found that spinal cord Hsp90 inhibition enhanced opioid anti-nociception in tail flick and paw incision pain at a single morphine dose^16^. While suggestive, these observations do not establish that the therapeutic index of morphine was improved, since we did not test a full dose range or any side effects in that earlier study. We thus hypothesized that spinal cord Hsp90 inhibition would consistently enhance morphine anti-nociception over a full dose range in different acute and chronic pain models while either reducing or not changing side effect potencies. We tested this using male and female CD-1 mice injected with 0.01 nmol of the non-isoform-selective Hsp90 inhibitor KU-32 (Fig. 1A) or Vehicle control by the i.t. route with a default treatment time of 24 h (dose and time point based on our earlier work^13–16^). After the 24-h treatment time, the mice were treated with a dose range of morphine and anti-nociception was measured.

We first tested acute thermal nociception in uninjured mice using the well-established tail flick model. KU-32 treatment caused a consistent and significant elevation in morphine anti-nociception over the dose range of 1–5.6 mg/kg (Fig. 1B–D). The response was not different at 10 mg/kg; however, 10 mg/kg is a maximal dose in this assay, and responses were not recorded past the 10 s threshold (Fig. 1E). Dose/response analysis revealed a potency of 3.0 (2.5–3.7; 95% confidence intervals reported in parentheses) mg/kg in Vehicle-treated mice and 1.6 (1.3–2.0) mg/kg in KU-32-treated mice, representing a 1.9-fold shift improvement in potency (Fig. 1F).

**Figure 2:**
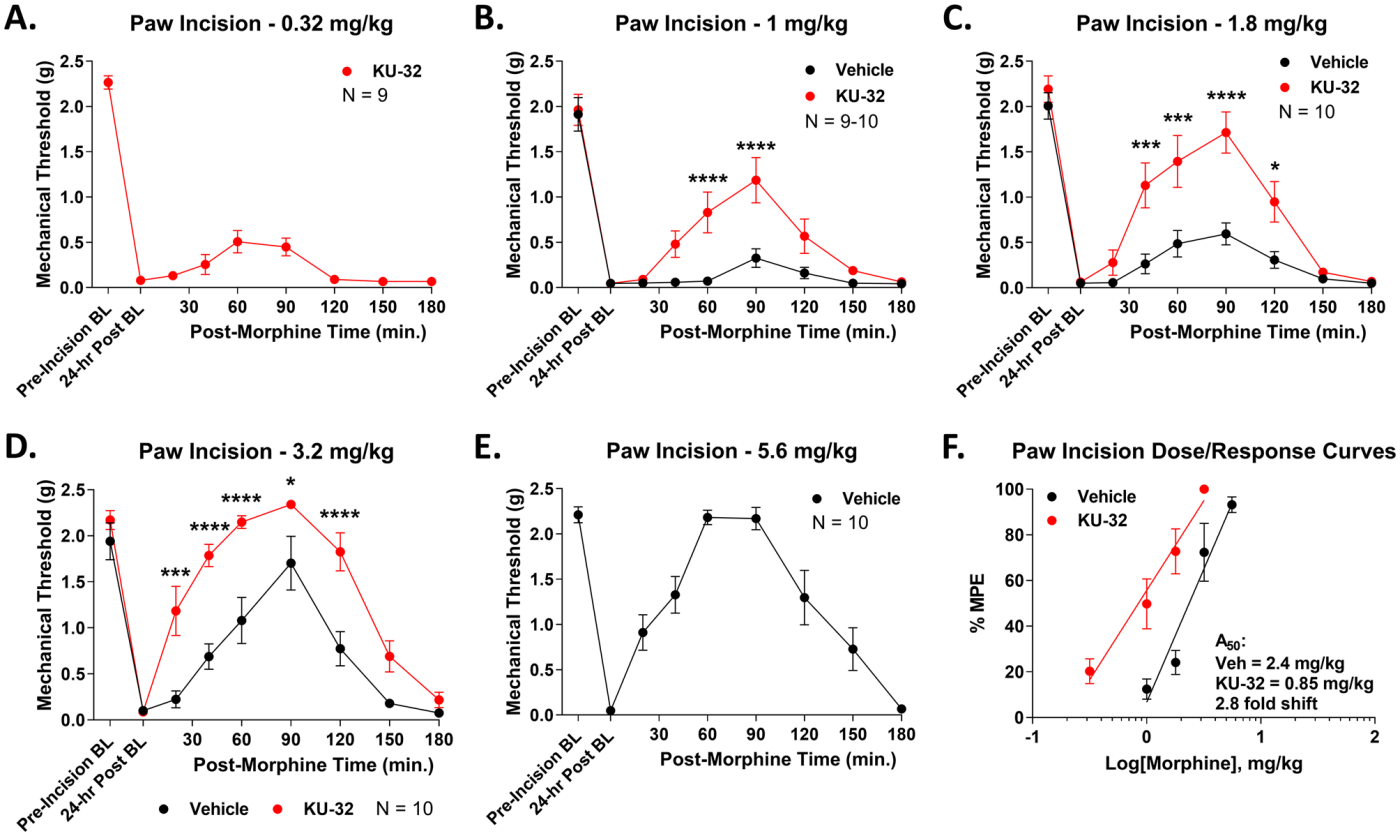
Spinal Hsp90 inhibition increases morphine potency in acute post-surgical paw incision pain. Male and female CD-1 mice had the paw incision surgery performed, then were injected i.t. with 0.01 nmol KU-32 or Vehicle control, 24 h, then 0.32–5.6 mg/kg morphine, s.c.. Paw withdrawal responses were recorded using von Frey filaments, including pre- and post-surgical baselines, validating the pain state. Males and females did not differ by 2 Way ANOVA (p > 0.05), so all male and female data were combined. The data were reported as the mean ± SEM, with sample sizes of mice/group noted in each graph. 2 technical replicates were performed per dose. *, ***, **** = p < 0.05, 0.001, 0.0001 vs. same time point Vehicle group by RM 2 Way ANOVA with Sidak’s post hoc test. (**A**–**E**) Individual dose curves shown as noted. The data were not normalized. Vehicle at 0.32 mg/kg and KU-32 at 5.6 mg/kg were not measured since these would be too low (Vehicle) or hit the assay threshold (KU-32), meaning they would not fit the linear dose/response model used. (**F**) Dose/response analysis was performed for individual curves, normalized as %MPE (baseline vs. 2.34 g cutoff), with linear regression for A_50_ calculation (see “Methods”). A_50_: Vehicle = 2.4 (2.0–2.8) mg/kg; KU-32 = 0.85 (0.64–1.1) mg/kg; 2.8 fold increase in potency.

This result was promising for potential translational benefit; however, tail flick is a spinal reflex in uninjured animals, and may not translate to clinical pain conditions. We thus used the post-surgical paw incision assay to model post-surgical pain, a common opioid indication^38^. Much as in heat-induced tail flick, KU-32 caused a significant and consistent elevation in morphine anti-nociception over the 0.32–5.6 mg/kg dose range (Fig. 2A–E). Dose/response analysis showed an A_50_ potency value of 2.4 (2.0–2.8) mg/kg for Vehicle animals and 0.85 (0.64–1.1) mg/kg for KU-32 animals, representing a 2.8-fold improvement in potency (Fig. 2F).

**Figure 3:**
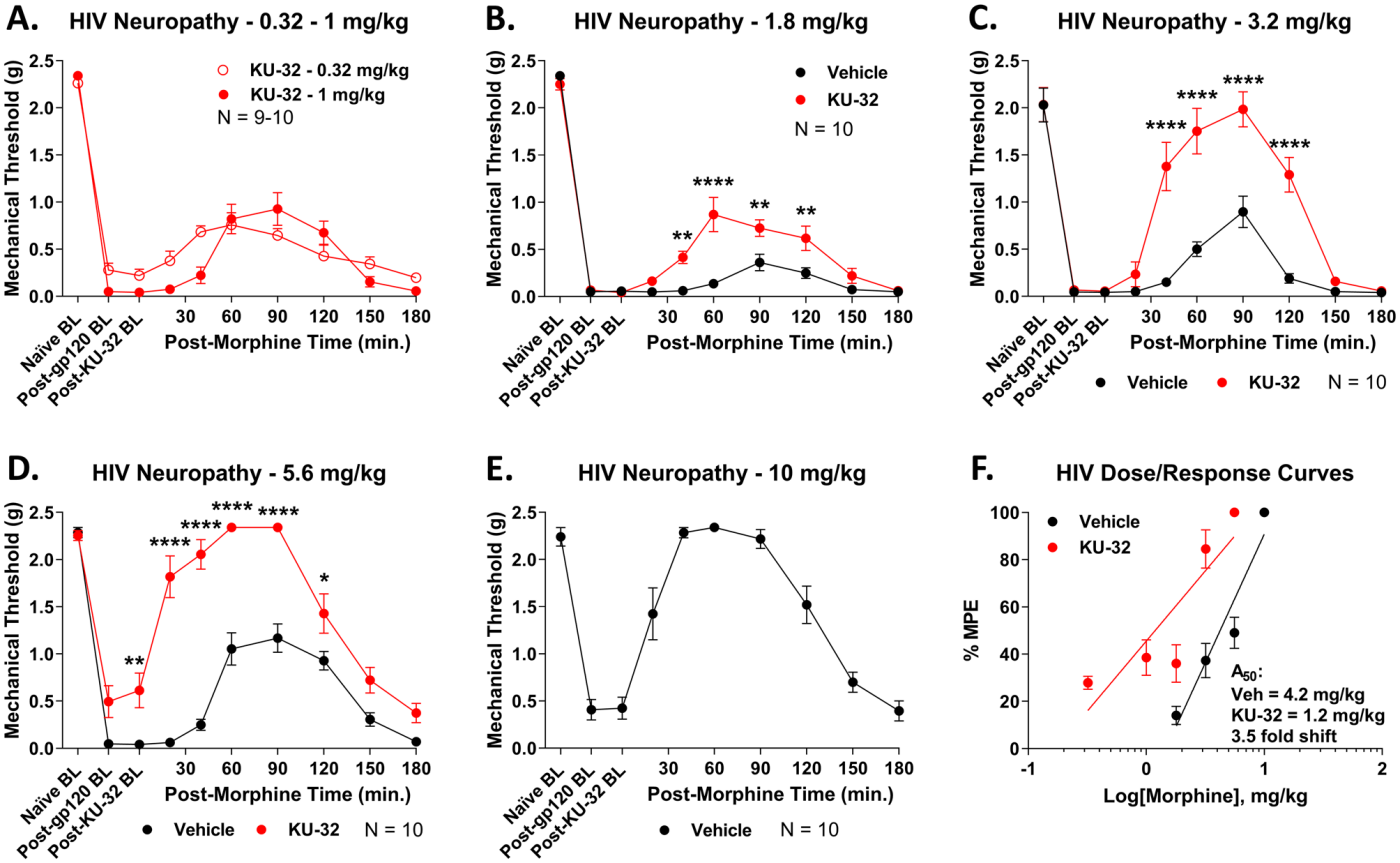
Spinal Hsp90 inhibition increases morphine potency in chronic HIV neuropathy pain. Male and female CD-1 mice were injected i.t. with gp120 protein on days 1, 3, and 5 (see Methods). Males and females did not differ by 2 Way ANOVA (p > 0.05), so all male and female data were combined. On day 20, mice were injected i.t. with 0.01 nmol KU-32 or Vehicle control, 24 h, then 0.32–10 mg/kg morphine, s.c.. Paw withdrawal responses were recorded using von Frey filaments, including pre- and post-treatment baselines, validating the pain state. The data were reported as the mean ± SEM, with sample sizes of mice/group noted in each graph. 2 technical replicates were performed per dose. *, **, **** = p < 0.05, 0.01, 0.0001 vs. same time point Vehicle group by RM 2 Way ANOVA with Sidak’s post hoc test. (**A**–**E**) Individual dose curves shown as noted. The data were not normalized. Vehicle at 0.32–1 mg/kg and KU-32 at 10 mg/kg were not measured since these would be too low (Vehicle) or hit the assay threshold (KU-32), meaning they would not fit the linear dose/response model used. (**F**) Dose/response analysis was performed for individual curves, normalized as %MPE (baseline vs. 2.34 g cutoff), with linear regression for A_50_ calculation (see “Methods”). A_50_: Vehicle = 4.2 (3.7–4.8) mg/kg; KU-32 = 1.2 (0.87–1.5) mg/kg; 3.5-fold increase in potency.

These results again suggest potential translational benefit, but both pain states are acute and short in duration, and do not represent chronic pain, which is the most difficult to treat in the clinic. We thus used the HIV peripheral neuropathy model induced by gp120 protein injection, which is a sustained and long-lasting neuropathic pain model^39^. After 3 sustained weeks of chronic pain, the mice were treated with KU-32 or Vehicle control and tested as above. Again, KU-32 treatment caused a sustained and significant increase in morphine anti-nociception over the 0.32–10 mg/kg dose range (Fig. 3A–E). Dose/response analysis showed a potency of 4.2 (3.7–4.8) mg/kg for Vehicle treatment and 1.2 (0.87–1.5) mg/kg for KU-32 treatment, an improvement of 3.5-fold (Fig. 3F). Not only did KU-32 improve opioid anti-nociception in this chronic pain model, it had the largest fold-shift improvement of the 3 models tested. Importantly, KU-32 treatment did not cause baseline differences in any pain state measured.

### Spinal Hsp90 inhibition reduces morphine tolerance and rescues established tolerance

**Figure 4:**
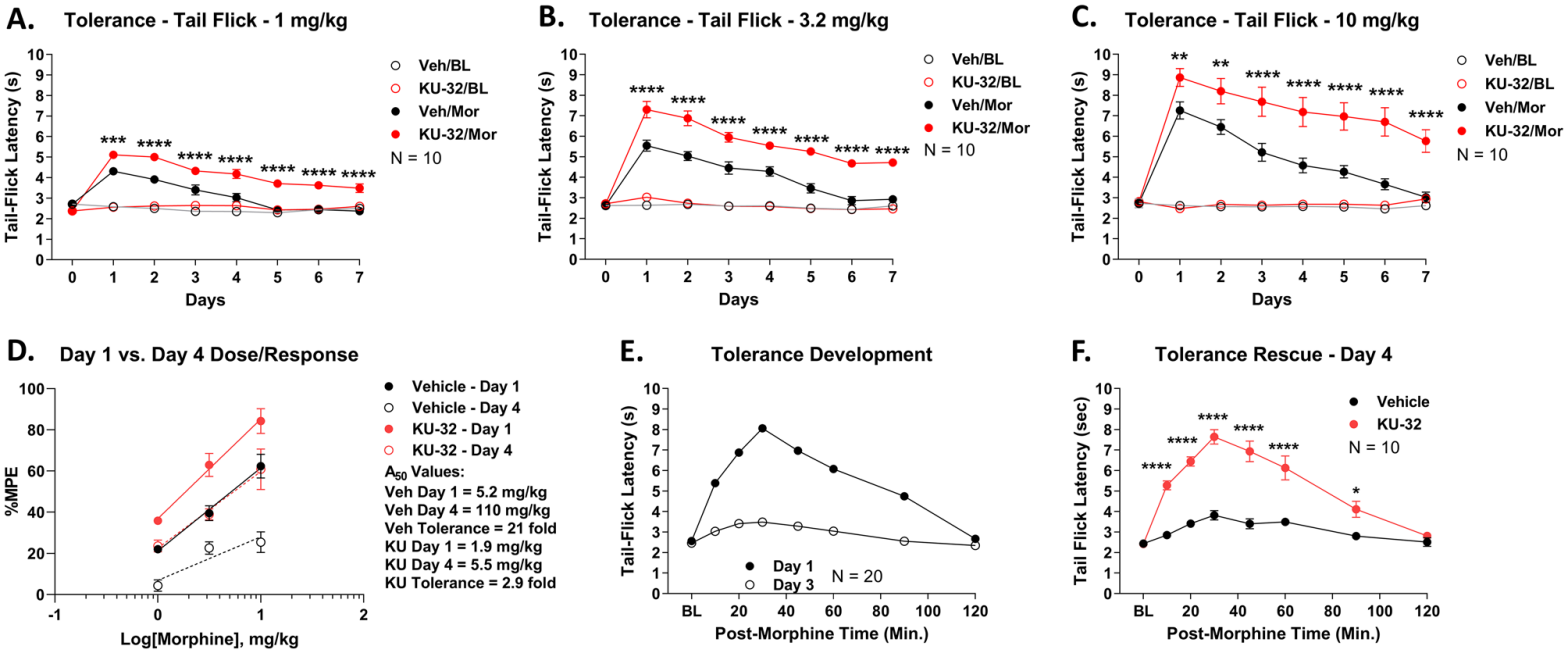
Spinal Hsp90 inhibition reduces morphine anti-nociceptive tolerance and rescues established tolerance. The data were reported as the mean ± SEM with sample sizes of mice/group noted in the graphs. Males and females did not differ by 2 Way ANOVA (p > 0.05), so all male and female data were combined. (**A**–**C**) Male and female CD-1 mice were injected with 0.01 nmol KU-32 or Vehicle control i.t. beginning on day 0 and continuing daily until day 6. The mice were injected with 1–10 mg/kg morphine s.c. twice daily beginning on day 1 and continuing daily until day 7. KU-32 injection preceded morphine injection each day. Thermal tail flick latencies (52 °C, 10 s cutoff) were recorded before treatment on day 0, daily before morphine injection (“BL”), and 30 min after each morning morphine injection on days 1–7. **, ***, **** = p < 0.01, 0.001, 0.0001 vs. same time point Veh/Mor group by RM 2 Way ANOVA with Tukey’s post hoc test. Individual dose/time curves shown as labeled. KU-32 treatment caused consistent elevation over Vehicle treatment over the 7-day period in all doses. The data were not normalized. (**D**) Day 1 vs. Day 4 dose/response analysis was performed with the data from (**A**–**C**). The data were normalized to %MPE (baseline vs. 10 s cutoff), with linear regression for A_50_ calculation (see “Methods”). A_50_: Vehicle Day 1 = 5.2 (3.9–7.5) mg/kg, Vehicle Day 4 = 110 (30–6300) mg/kg, 21 fold shift; KU-32 Day 1 = 1.9 (1.3–2.5) mg/kg, KU-32 Day 4 = 5.5 (3.5–12) mg/kg, 2.9 fold shift. (**E**) Male and female mice were treated with twice daily morphine (10 mg/kg, s.c.) for 3 days to establish tolerance in all mice. Tail flick responses shown on day 1 (acute morphine) and day 3 (morphine-tolerant). (**F**) On day 3, after measuring the tail flick time course, the mice were injected with 0.01 nmol KU-32 or Vehicle control, i.t., with a 24-h recovery. On day 4, the mice were injected again with 10 mg/kg morphine, s.c., and a tail-flick time course performed. Experiment performed in 2 technical replicates. *, **** = p < 0.05, 0.0001 vs. same time point Vehicle group by RM 2 Way ANOVA with Sidak’s post hoc test. The KU-32 treated mice, previously tolerant on day 3, showed a statistically significant increase in morphine anti-nociception when compared to Vehicle.

The results above support our hypothesis that spinal Hsp90 inhibition could make opioids more potent. However, at the same time, if side effect potencies are enhanced, then the improvement does not result in an improved therapeutic index or improved opioid therapy. We thus tested the potency of morphine side effects with spinal Hsp90 inhibitor treatment, beginning with anti-nociceptive tolerance. Over a 7-day repeated treatment period, the anti-nociceptive efficacy of morphine steadily decreased in Vehicle-treated mice, resulting in a complete loss of anti-nociceptive efficacy by day 7 for the entire 1–10 mg/kg dose range (Fig. 4A–C). In contrast, KU-32 treatment caused a significant decrease in tolerance over the full 7-day treatment period and full dose range, so that at least some significant anti-nociceptive efficacy remained by day 7 at each dose with KU-32 treatment (Fig. 4A–C). Notably, baseline responses were not altered by any treatment, demonstrating that KU-32 treatment is not altering baseline nociception (Fig. 4A–C). To quantify this result, we compared day 1 vs. day 4 responses for each dose and treatment; day 4 was chosen because days 5–7 have no quantifiable Vehicle response for at least one dose each. Dose/response analysis of these data revealed a 21-fold tolerance shift from day 1 to day 4 for Vehicle-treated mice; this tolerance shift was reduced to 2.9-fold in KU-32-treated mice (Fig. 4D).

This result suggests that morphine tolerance is blocked by KU-32 treatment; however, KU-32 treatment began before the tolerance regimen and continued during every morphine injection. We thus sought to determine if KU-32 treatment would reverse already-established tolerance. We thus subjected naïve mice to a tolerance regimen as above for 3 days; all mice showed normal morphine anti-nociception on day 1 and a near-complete tolerance by day 3 (Fig. 4E). We then injected these mice with KU-32 or Vehicle, and 24 h later, injected morphine again. The Vehicle-treated mice showed little anti-nociception, showing how their tolerance was maintained (Fig. 4F). In sharp contrast, the KU-32-treated mice showed a full anti-nociceptive response to morphine, comparable to their day 1 response (Fig. 4F). These results suggest that not only can spinal Hsp90 inhibition reduce the development of tolerance, it can restore responsiveness to already-tolerant mice.

### Spinal Hsp90 inhibition does not alter morphine-induced constipation and reward

**Figure 5:**
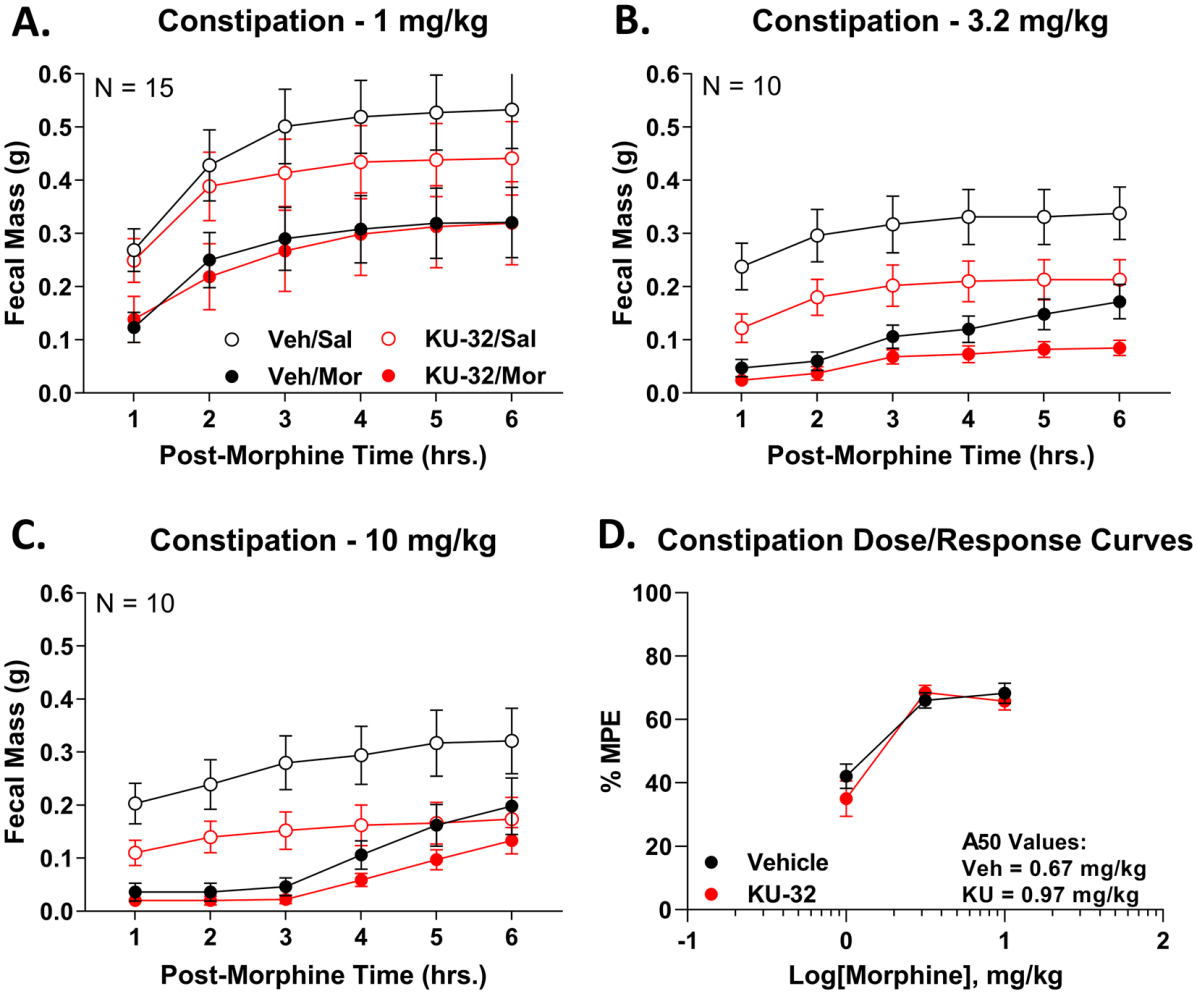
Spinal Hsp90 inhibition does not alter morphine-induced constipation. Male and female CD-1 mice were treated with 0.01 nmol KU-32 or Vehicle control i.t., 24 h, followed by 1–10 mg/kg morphine, s.c.. Fecal mass was measured over 6 h post-morphine, and used to construct cumulative plots. Males and females did not differ by 2 Way ANOVA (p > 0.05), so all male and female data were combined. Curves for morphine as well as saline-injected controls shown. The data were reported as the mean ± SEM with the sample size of mice/group noted in the graphs. Experiments were performed in 2–3 technical replicates. (**A**–**C**) Individual dose curves reported, along with saline-injected controls. KU-32-treated saline or morphine groups were not statistically different from Vehicle-, saline- or morphine-injected groups, respectively, at any time point (p > 0.05). (**D**) Morphine-injected animals were normalized to saline-injected controls for each treatment group (Vehicle or KU-32), and used to construct dose/response curves. The 6-h area under the curve (AUC) data from (**A**–**C**) were further normalized to %MPE (100% MPE = 0% fecal production/100% constipation), with linear regression for A_50_ calculation (see “Methods”). Only the 1 and 3.2 mg/kg dose curves were used to calculate the A_50_ since the dose response plateaus between 3.2 and 10 mg/kg. A_50_: Vehicle = 0.67 (0.32–0.94) mg/kg; KU-32 = 0.97 (0.59–1.3) mg/kg.

Based on our rationale described above, we continued to investigate clinically relevant opioid side effects. Opioid-induced constipation is a highly clinically significant side effect, that lowers medication compliance and patient quality of life^18^. After KU-32 or Vehicle treatment, we injected a dose-range of morphine and measured fecal output over a 6-h time course. Compared to saline-injected controls, morphine caused ~ 40% constipation at 1 mg/kg, that plateaued at ~ 70% constipation at 3.2–10 mg/kg (Fig. 5A–C). Vehicle vs. KU-32 saline or morphine treatment curves were not significantly different at any dose or time point. After normalizing each treatment group to saline-injected controls, dose/response analysis revealed overlapping curves with a constipation potency of 0.67 (0.32–0.94) mg/kg for Vehicle and 0.97 (0.59–1.3) mg/kg for KU-32, further supporting the conclusion that KU-32 treatment did not alter morphine constipation (Fig. 5D).

**Figure 6:**
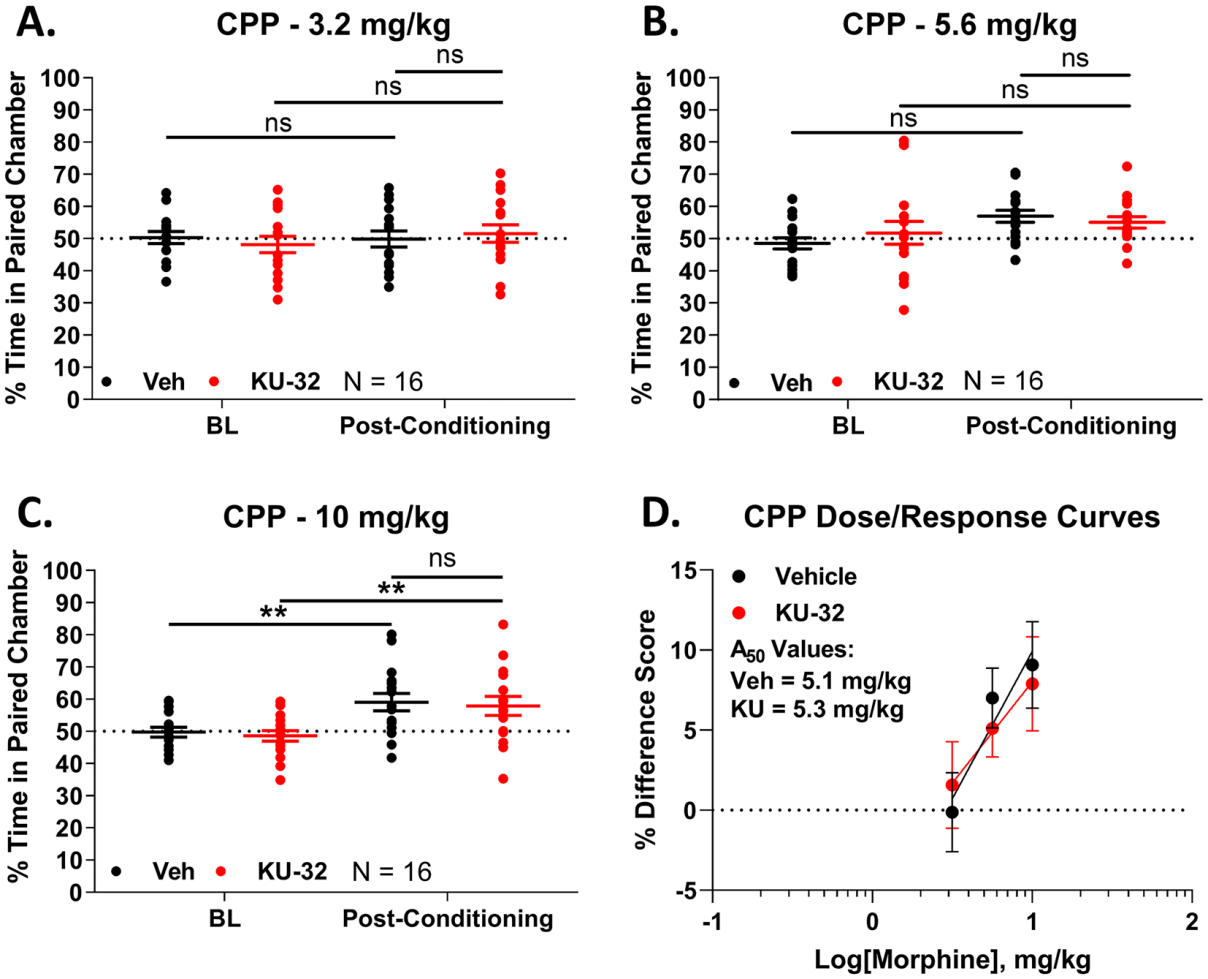
Spinal Hsp90 inhibition does not alter reward learning. Male and female mice were treated over 4 days with KU-32 or Vehicle treatment i.t., combined with 3.2–10 mg/kg morphine, s.c. as a conditioned place preference (CPP) stimulus (see Methods). Males and females did not differ by 2 Way ANOVA (p > 0.05), so all male and female data were combined. On day 5, paired chamber preference was recorded. The data were reported as the mean ± SEM with the sample size of mice/group noted in the graphs. Each dose was performed in 2 technical replicates. ** = p < 0.01 vs. same treatment Baseline (BL) by RM 2 Way ANOVA with Tukey’s post hoc test. (**A**–**C**) The individual doses are shown. There was a trend to increasing preference with increasing dose that reached significance over baseline at 10 mg/kg for both Vehicle and KU-32 treatment. There was no difference between Vehicle and KU-32 treatment (p > 0.05). (**D**) Dose/response analysis was performed, with the data reported as the % Difference Score (see “Methods”), with linear regression for A_50_ calculation (see Methods). For the purposes of A_50_ calculation, the response at 10 mg/kg in Vehicle-treated mice was considered to be a maximal response, since 100% preference is not possible in this assay. A_50_: Vehicle = 5.1 (2.7–7.7) mg/kg; KU-32 = 5.3 (− ∞– + ∞) mg/kg.

We next tested opioid-induced reward, which is the basis for opioid addiction, and has contributed to an opioid abuse and overdose crisis^17,40^. We used the well-established conditioned place preference (CPP) assay, which demonstrates reward (or aversion) learning^26^. Over the 3.2–10 mg/kg morphine dose range, we observed an increasing preference for the morphine-paired chamber by Vehicle-treated mice, that rose to the level of significance at 10 mg/kg of morphine (Fig. 6A–C). KU-32 treatment did not differ significantly from Vehicle treatment at any dose, and also showed significant preference at 10 mg/kg of morphine (Fig. 6A–C). Dose/response analysis showed overlapping curves and a potency of 5.1 (2.7–7.7) mg/kg for Vehicle and 5.3 (− ∞–+ ∞) mg/kg for KU-32, further supporting a lack of effect of KU-32 treatment on morphine-induced reward learning (Fig. 6D).

### Identification of spinal cord-specific Hsp90 isoforms

The results above confirm our basic hypothesis that inhibition of Hsp90 in the spinal cord can improve the therapeutic index of opioids. However, all experiments above were performed with i.t. injection, which is of limited therapeutic relevance, and we already know that systemic delivery of non-selective Hsp90 inhibitors blocks opioid anti-nociception by inhibiting active Hsp90 in the brain^16^. We thus sought a way to block spinal Hsp90 in a clinically relevant way. In our earlier work, we found that of the 4 Hsp90 isoforms, only Hsp90α was active in regulating opioid signaling in the brain^14^. We thus hypothesized that if different Hsp90 isoforms were active in the spinal cord, these could be targeted by systemically delivered *selective* inhibitors to selectively block spinal cord Hsp90.

**Figure 7:**
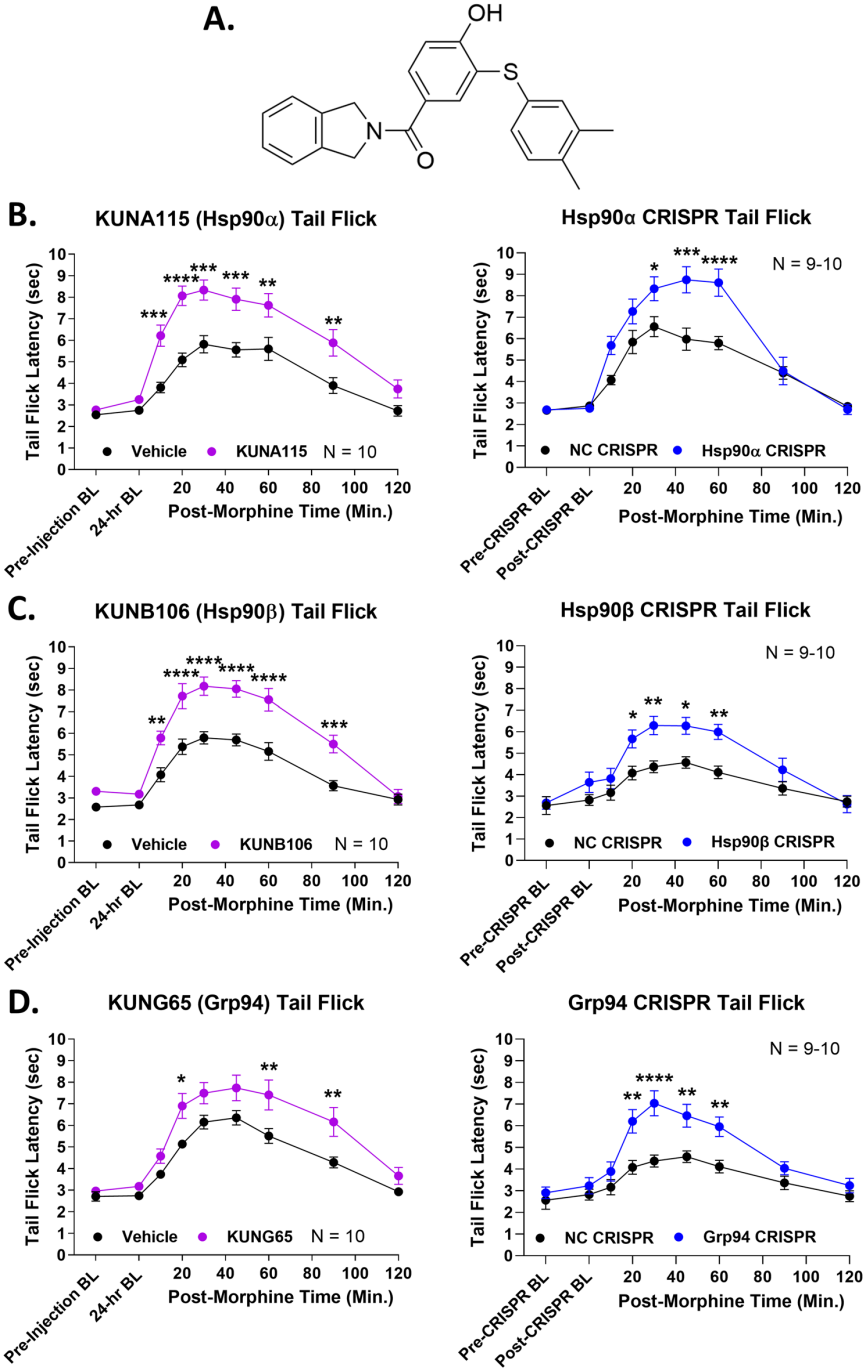
Identification of spinal-cord specific Hsp90 isoforms that regulate opioid anti-nociception. Male and female CD-1 mice were treated with 0.01 nmol of isoform-selective inhibitor or Vehicle control i.t. with a 24 h treatment time; or with an isoform-selective CRISPR knockdown construct or universal negative control CRISPR construct (NC) with a 10-day treatment time (see “Methods”). Males and females did not differ by 2 Way ANOVA (p > 0.05), so all male and female data were combined. Pre- and post-treatment baselines were recorded using the 52 °C tail flick assay (as above), as well as tail flick timecourses in response to 3.2 mg/kg morphine s.c. The data were presented as the mean ± SEM with the sample size of mice/group noted in each graph. Each experiment was completed in 2 technical replicates. *, **, ***, **** = p < 0.05, 0.01, 0.001, 0.0001 vs. same time point Vehicle/NC group by 2 Way RM ANOVA with Sidak’s post hoc test. The Hsp90α isoform was targeted with the selective inhibitor KUNA115 (**A**) as well as selective CRISPR (**B**). (**C**) The Hsp90β isoform was targeted with the selective inhibitor KUNB106 as well as selective CRISPR. (**D**) The Grp94 isoform was targeted with the selective inhibitor KUNG65 as well as selective CRISPR. Both methods caused significant anti-nociceptive elevation for all 3 isoforms, confirming that all 3 isoforms regulate opioid anti-nociception in the spinal cord.

We thus treated mice with selective small molecule inhibitors and targeted CRISPR constructs for each Hsp90 isoform, all delivered by the i.t. route. We found that the Hsp90α-selective inhibitor KUNA115 (Fig. 7A) and Hsp90α-targeted CRISPR enhanced morphine anti-nociception in the tail flick assay (Fig. 7B). This result suggests that Hsp90α regulates opioid signaling in the brain *and* the spinal cord. However, unlike in the brain, inhibitors and CRISPR targeted to Hsp90β and Grp94 also enhanced morphine pain relief (Fig. 7C,D). This outcome suggests that all 3 Hsp90 isoforms regulate opioid signaling in the spinal cord, while only Hsp90α is active in the brain. If our hypothesis is correct, then systemic inhibition of Hsp90β and Grp94 should recapitulate the benefits of Hsp90 inhibition in the spinal cord by the i.t. route.

### Systemic Grp94 inhibition recapitulates the benefits of spinal Hsp90 inhibition

**Figure 8:**
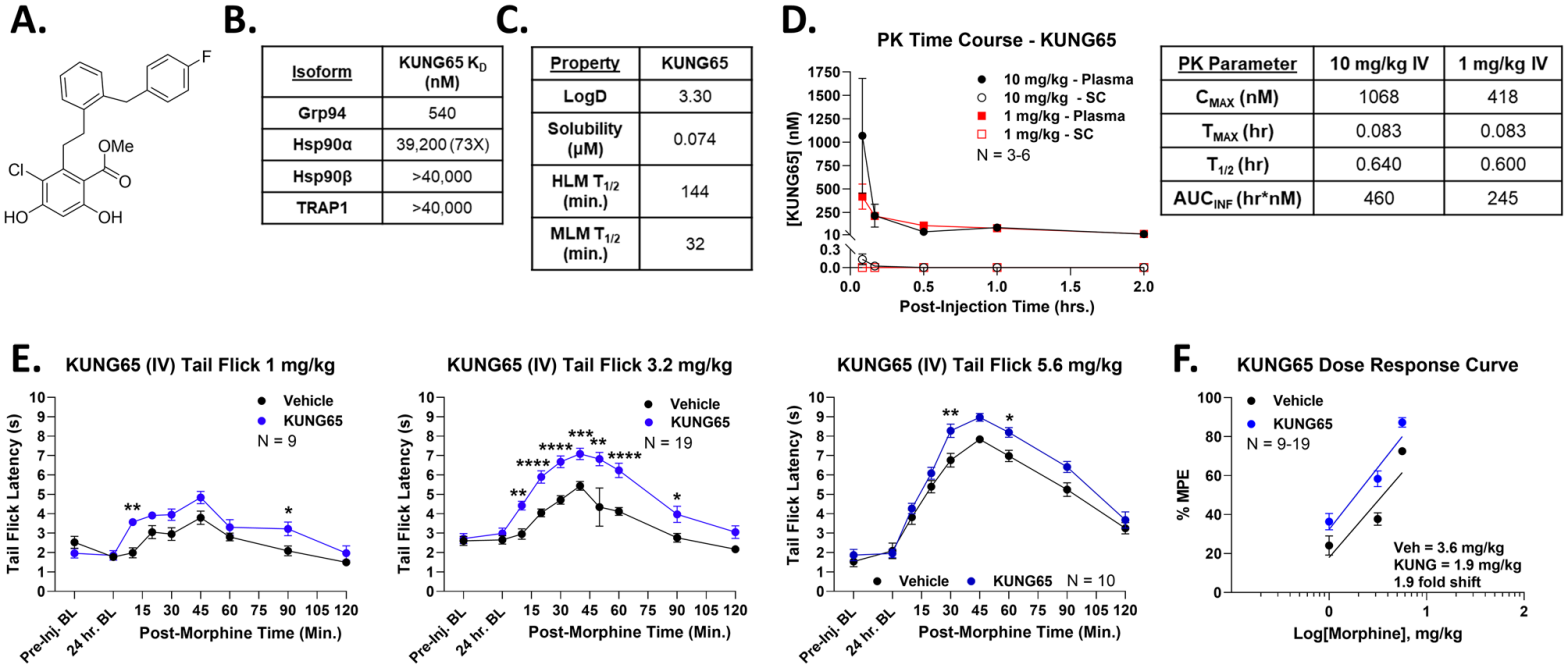
Systemic Grp94 inhibition enhances morphine anti-nociception. (**A**) Chemical structure of KUNG65. (**B**) The affinity (K_D_) of KUNG65 for each Hsp90 isoform is shown; KUNG65 has a minimum selectivity of 73-fold vs. other isoforms (data taken from Ref.^41^). (**C**) In vitro ADME parameters for KUNG65 are shown (see Methods). HLM/MLM = human/mouse liver microsomes; T_1/2_ = half-life. (**D**) A pharmacokinetic (PK) study with KUNG65 was performed, dosed at 1–10 mg/kg by the i.v. route in male and female CD-1 mice (see Methods). Males and females did not differ by 2 Way ANOVA (p > 0.05), so all male and female data were combined. The data were presented as the mean ± SEM, with the sample size of mice/group noted in the graphs, performed in one technical replicate. The KUNG65 is detectable in both plasma and spinal cord, with PK parameters for i.v. dosing shown in the attached table. These results are sufficient to show that the drug has systemic exposure and low blood–brain barrier penetration with i.v. dosing. (**E**) Male and female CD-1 mice were treated with KUNG65 (1 mg/kg) or Vehicle injected i.v. with a 24 h treatment time, followed by 1–5.6 mg/kg morphine, s.c., with tail flick time courses performed. The data were presented as the mean ± SEM, with the sample size of mice/group noted in the graphs, performed in 2—4 technical replicates. *, **, ***, **** = p < 0.05, 0.01, 0.001, 0.0001 vs. same time point Vehicle group by 2 Way RM ANOVA with Sidak’s post hoc test. Morphine anti-nociception is consistently elevated, similar to the results with direct spinal inhibition above in Fig. 1F). The data were transformed into peak %MPE and used to construct dose/response curves, with linear regression for A_50_ calculation (see “Methods”). Vehicle = 3.6 (2.9–4.6) mg/kg, KUNG65 = 1.9 (1.4–2.3) mg/kg; 1.9-fold improvement in morphine potency.

To test this hypothesis, we used the selective Grp94 inhibitor KUNG65 (Ref.^23^, Fig. 8A). KUNG65 is a strongly selective Grp94 inhibitor, with a target K_D_ of 540 nM and at least 73-fold selectivity vs. the other Hsp90 isoforms (Fig. 8B), making this molecule a good choice for this study. However, we are the first to use KUNG65 in vivo, and systemic delivery requires metabolic stability and the ability to cross the blood–brain barrier, which is unknown for this molecule. We thus tested in vitro ADME parameters of KUNG65, finding a LogD of 3.3, a solubility of 0.074 μM, and a metabolic half-life of 144 and 32 min in human and mouse liver microsomes, respectively (Fig. 8C). While the solubility is low, the other parameters were promising for in vivo delivery. We thus performed a pharmacokinetic study, giving KUNG65 at 1 and 10 mg/kg by the intravenous route. We found strong KUNG65 levels in the plasma, peaking at 418 nM (1 mg/kg) and 1068 nM (10 mg/kg), and very low exposure in the spinal cord (Fig. 8D). While KUNG65 had relatively poor pharmacokinetic performance, this result was sufficient to demonstrate that low levels of the drug could penetrate into the site of action in the spinal cord. We thus delivered KUNG65 at 1 mg/kg by the i.v. route in a similar experimental design to the pain assays above, followed by a morphine dose–response in the tail flick assay. Much like our results with KU-32 above, we found that i.v. KUNG65 consistently elevated tail flick anti-nociception in response to morphine across the 1–5.6 mg/kg dose range (Fig. 8E). We constructed a dose–response curve, finding an A_50_ of 3.6 (2.9–4.6) mg/kg in Vehicle-treated mice, and 1.9 (1.4–2.3) mg/kg in the KUNG65-treated mice, a 1.9-fold shift improvement in morphine potency (Fig. 8F). This 1.9-fold shift is identical to the fold shift found with i.t. KU-32 treatment in Fig. 1, providing a strong initial confirmation of our hypothesis.

To further evaluate the therapeutic potential of KUNG65, we tested tail flick anti-nociception 48 h after treatment, instead of 24 h as above. This experiment found no effect of KUNG65 treatment, suggesting the effects of the drug persist for 24 but not 48 h (Fig. S1). To rule out confounding effects at the opioid receptors, we also tested for the ability of KUNG65 to bind to any of the human opioid receptors, finding no binding up to a 10 μM concentration (Fig. S2). In a further control, since spinal cord exposure was low, we knocked down Grp94 expression in the spinal cord using CRISPR prior to systemic i.v. injection of KUNG65 (Fig. S3). In this experiment, we observed that spinal cord Grp94 knockdown abolished the benefit of systemic KUNG65, suggesting the drug can engage the target in the spinal cord. We also tested a higher 10 mg/kg dose of KUNG65, and found that while it elevated morphine pain relief, it did so less than a 1 mg/kg dose, suggesting that 1 mg/kg is near the top of the dose curve (Fig. S4). Lastly, we found that KUNG65-enhanced morphine pain relief was fully naloxone-reversible, suggesting that the benefits of Hsp90-enhanced pain relief are fully carried out by the opioid system (Fig. S5).

Continuing our opioid anti-nociception analysis, we then tested i.v. KUNG65 in the post-surgical paw incision model. As expected, i.v. KUNG65 significantly elevated the morphine anti-nociceptive response across the 1–3.2 mg/kg dose range (Fig. S6A). Dose–response analysis showed an A_50_ of 2.0 (1.4–3.0) mg/kg in Vehicle-treated mice and 0.9 (0.5–1.2) mg/kg in the KUNG65-treated mice, a 2.2-fold shift improvement in morphine potency (Fig. S6B). Again, these results support our hypothesis that systemic selective inhibition of Grp94 can mimic the effects of spinal cord Hsp90 inhibition.

### Systemic Hsp90β inhibition recapitulates the benefits of spinal Hsp90 inhibition

**Figure 9:**
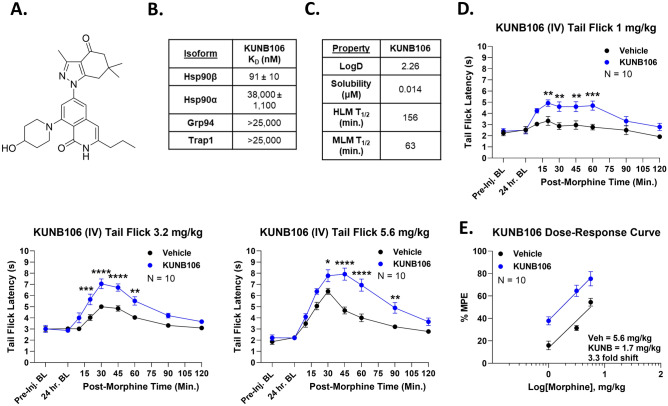
Systemic Hsp90β inhibition enhances morphine anti-nociception. (**A**) Chemical structure of the Hsp90β-selective inhibitor KUNB106. (**B**) Affinity (K_D_) of KUNB106 for each Hsp90 isoform, showing a minimum selectivity of 275 fold (data taken from Ref.^22^). (**C**) In vitro ADME parameters for KUNB106 are shown (see Methods). HLM/MLM = human/mouse liver microsomes; T_1/2_ = half-life. (**D**) Male and female CD-1 mice were treated with 1 mg/kg KUNB106 or Vehicle i.v., 24 h, followed by 1–5.6 mg/kg morphine s.c. and tail flick timecourses recorded. Males and females did not differ by 2 Way ANOVA (p > 0.05), so all male and female data were combined. The data were shown as the mean ± SEM, with the sample size of mice per group noted in each graph, completed in 2 technical replicates. *, **, ***, **** = p < 0.05, 0.01, 0.001, 0.0001 vs. same time point Vehicle group by 2 Way RM ANOVA with Sidak’s post hoc test. KUNB106 caused increased anti-nociception over the whole morphine dose range. (**E**) Dose–response curves were generated from the data in (**D**), after transformation to peak %MPE, with linear regression for A_50_ calculation (see “Methods”). A_50_: Vehicle = 5.6 (4.4.–∞) mg/kg, KUNB106 = 1.7 (1.1–2.3) mg/kg; 3.3-fold increase in morphine potency.

We continued to test our hypothesis, this time using the Hsp90β-selective inhibitor KUNB106 (Ref.^22^, Fig. 9A). Similar to KUNG65 above, KUNB106 is a highly selective Hsp90β inhibitor, with a target K_D_ of 91 nM and a minimum 275-fold selectivity vs. other isoforms (Fig. 9B). In vitro ADME analysis showed a LogD of 2.26, a solubility of 0.014 μM, and a human and mouse liver microsome half-life of 156 and 63 min, respectively (Fig. 9C). These ADME results and profile are similar to KUNG65 above, so we proceeded directly to in vivo testing, using the same 1 mg/kg i.v. dose and route as for KUNG65. Again, KUNB106 caused an enhanced morphine tail flick anti-nociceptive response across the entire 1–5.6 mg/kg dose range (Fig. 9D). Upon dose–response analysis, we found an A_50_ of 5.6 (4.4–∞) mg/kg for Vehicle-treated mice and 1.7 (1.1–2.3) mg/kg for KUNB106-treated mice, a 3.3-fold shift (Fig. 9E). As a test for potential confounds, we tested for the ability of KUNB106 to bind to the opioid receptors, which could explain these results. We did not find any binding to any opioid receptor up to a 10 μM concentration, suggesting our findings are on-target to Hsp90β inhibition (Fig. S2). In further control experiments, similar to KUNG65 above, we found that 10 mg/kg KUNB106 produced less benefit than 1 mg/kg (Fig. S4), and that KUNB106-enhanced morphine pain relief was fully naloxone-reversible (Fig. S5).

Extending this analysis again to the post-surgical paw incision model, we found that KUNB106 elevated morphine anti-nociception across the 1–3.2 mg/kg dose range (Fig. S7A). We found an A_50_ of 2.5 (2.0–∞) mg/kg for Vehicle-treated mice and 0.99 (∞–∞) mg/kg for KUNB106-treated mice, a 2.5-fold shift (Fig. S7B). Together these results support our hypothesis, in that systemic Hsp90β and Grp94 inhibitors both boosted morphine pain relief in a similar manner to direct spinal injection of a non-selective inhibitor like KU-32, suggesting that they are targeting active Hsp90 isoforms in the spinal cord and avoiding active Hsp90 isoforms in the brain.

### Systemic Grp94 and Hsp90β inhibitors improve the therapeutic index of morphine

The above results establish that systemic inhibition of Grp94 and Hsp90β boost pain relief much like we saw with intrathecal injection of Hsp90 inhibitor. However, these results do not show that systemic Grp94/Hsp90β inhibitors either improve or do not change side effects, which is necessary to fully show that these treatments boost the therapeutic index of morphine. We thus tested for the impact of KUNG65 and KUNB106 on tolerance and respiratory depression, a key side effect linked to opioid safety and overdose.

**Figure 10:**
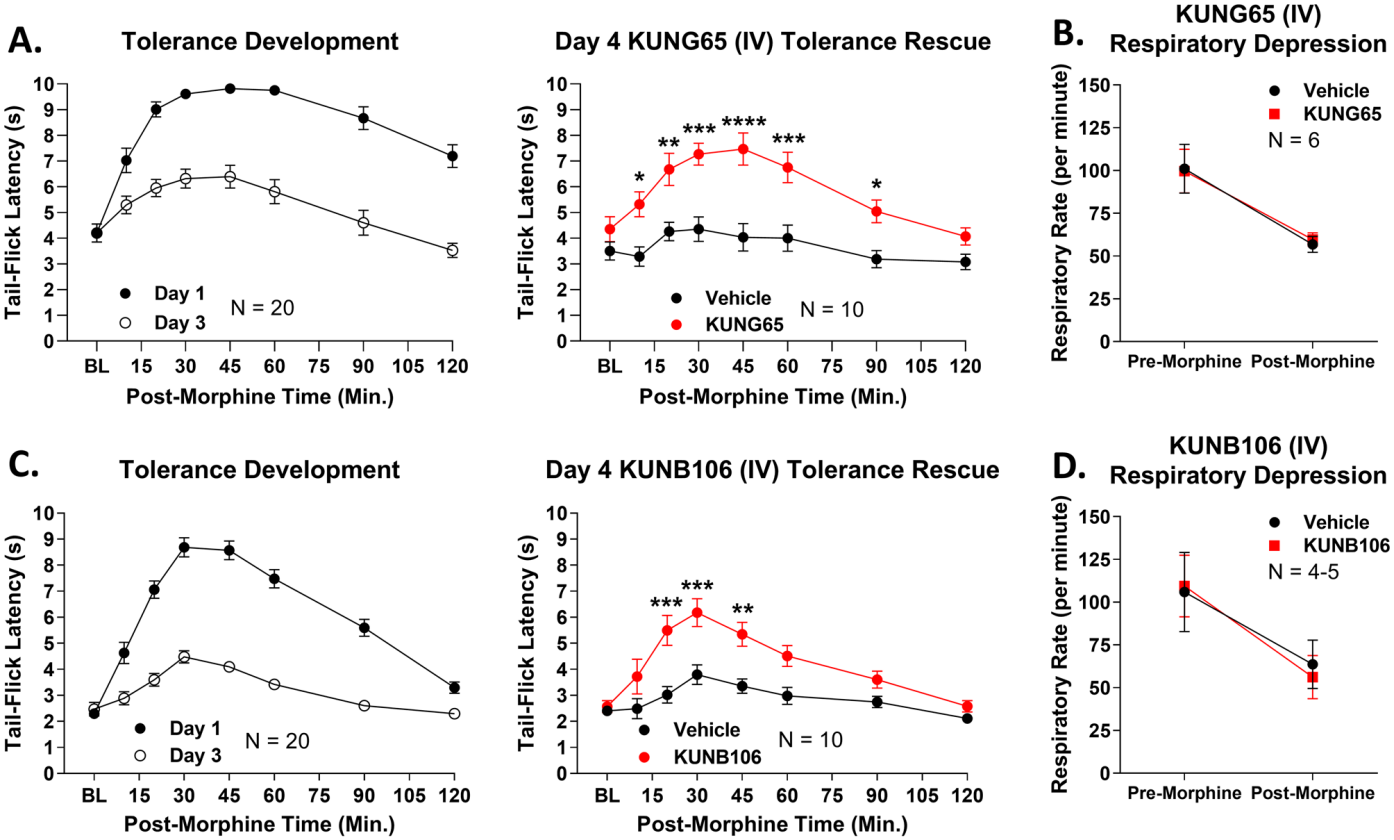
Systemic Grp94 and Hsp90β inhibition rescues established tolerance without worsening opioid-induced respiratory depression. Male and female CD-1 mice used for every experiment, with data presented as the mean ± SEM and the sample size of mice/group noted in each graph. Males and females did not differ by 2 Way ANOVA (p > 0.05), so all male and female data were combined. The tolerance experiments were performed in 2 technical replicates, and the respiratory depression experiments in 1 technical replicate. *, **, ***, **** = p < 0.05, 0.01, 0.001, 0.0001 vs. same time point Vehicle group by 2 Way RM ANOVA with Sidak’s post hoc test. (**A**) Tolerance was induced in all mice over 3 days with twice daily injection of 10 mg/kg morphine s.c. (as in Fig. 4). On day 3, mice were injected with 1 mg/kg KUNG65 or Vehicle i.v., 24 h, followed by 10 mg/kg morphine s.c. and another tail flick time course. KUNG65 rescued established tolerance much like intrathecal injection of KU-32 above. (**B**) Mice were injected with 1 mg/kg KUNG65 or Vehicle i.v., 24 h, then habituated and baselined in a whole-body plethysmography chamber for 30 min (see Methods). All mice were then injected with 7.5 mg/kg morphine i.v., and respiratory activity recorded for another hour. KUNG65 had no effect on respiration before or after morphine injection (p > 0.05). (**C**) Tolerance was induced as above, and on day 3, 1 mg/kg KUNB106 or Vehicle injected i.v., 24 h, followed by 10 mg/kg morphine s.c. and another tail flick timecourse. KUNB106 also rescued established tolerance like systemic KUNG65 or intrathecal KU-32. (**D**) Mice were injected with 1 mg/kg KUNB106 or Vehicle i.v., 24 h, then respiratory activity measured as above (including 7.5 mg/kg morphine i.v. challenge). KUNB106 had no impact on respiratory activity before or after morphine (p > 0.05).

We first tested the Grp94 inhibitor KUNG65. We found that acute injection i.v. of 1 mg/kg KUNG65 could rescue established morphine tolerance (Fig. 10A). This finding was very similar to the tolerance rescue observed with spinal injection of KU-32 in Fig. 4E,F above and suggests that KUNG65 can prevent or rescue opioid tolerance. In respiratory depression, we found that KUNG65 had no effect on respiratory activity, either before or after morphine injection (Fig. 10B). This was an important safety finding, suggesting that while these inhibitors can boost opioid pain relief, they will not boost side effects, thus improving the therapeutic index of morphine. We found near-identical results for KUNB106, finding that KUNB106 injection rescued established tolerance (Fig. 10C) while also having no effect on morphine-induced respiratory depression (Fig. 10D). Together these results confirm that Hsp90β and Grp94 inhibitors do indeed boost the therapeutic index of morphine, enhancing pain relief while either improving or not changing side effects.

### Systemic Hsp90α inhibitor blocks opioid anti-nociception

Finally, we tested the last piece of our hypothesis by systemic i.v. injection of 1 mg/kg KUNA115, a selective Hsp90α inhibitor. If our hypothesis is correct, then this drug should inhibit active Hsp90 in both the brain and spinal cord, leading to a loss of anti-nociception as we saw with systemic non-selective inhibitor. We used this drug in the post-surgical paw incision model and found that this treatment completely blocked anti-nociception in response to 3.2 mg/kg morphine (Fig. S8). This last experiment ties together our model, finding that systemic Hsp90β and Grp94 inhibition recapitulates the beneficial effects of spinal cord inhibition, while Hsp90α inhibition recapitulates the negative effects of brain inhibition.

## Discussion

**Figure 11:**
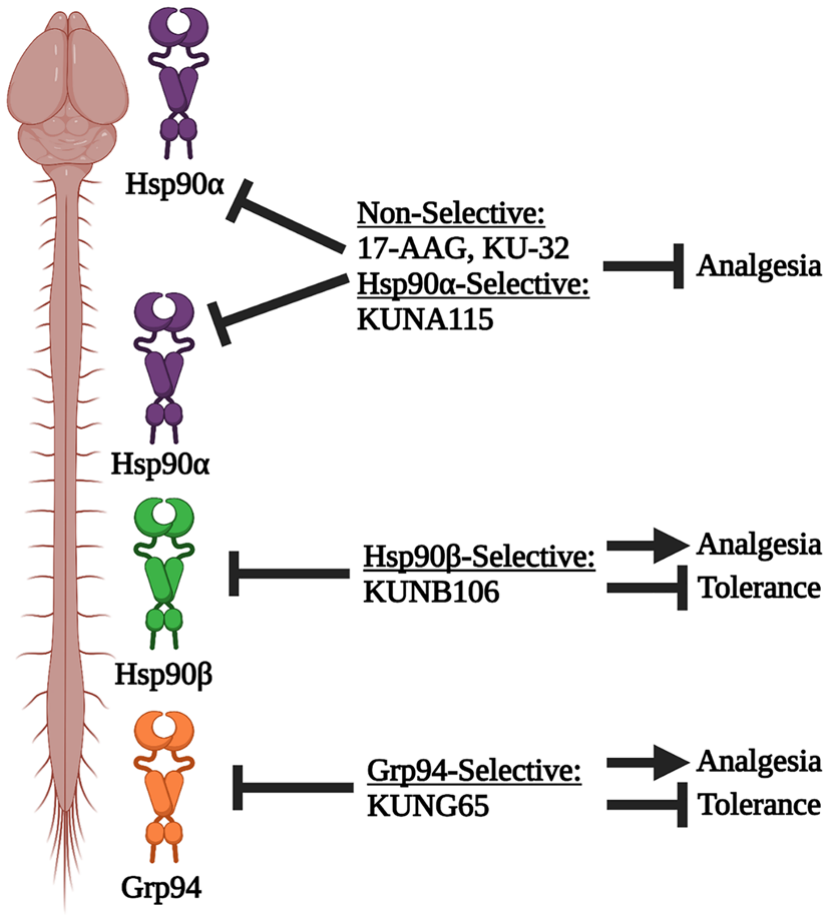
Model of Hsp90 isoform inhibition in the brain vs. spinal cord. In the brain, Hsp90α alone regulates opioid signaling, while in the spinal cord, Hsp90α, Hsp90β, and Grp94 all do so. When Hsp90α is inhibited systemically with non-selective inhibitor or Hsp90α-selective inhibitor, opioid anti-nociception is blocked, since brain Hsp90 is inhibited and a brain-like response is evoked. In contrast, when Hsp90β or Grp94 are inhibited systemically with selective inhibitors, brain inhibition of Hsp90α is avoided, and a spinal cord-like response is evoked—increased anti-nociception and decreased side effects. This model suggests that Hsp90β and Grp94 inhibitors could be given by translationally-relevant routes to improve the therapeutic index of opioids and enable a dose-reduction strategy. Figure created using biorender.com.

We show here that Hsp90 inhibition in the spinal cord improves the therapeutic index of morphine, by increasing anti-nociceptive potency by 2–fourfold, reducing and rescuing tolerance, while not changing reward and constipation potency. We have further uncovered a novel strategy to avoid unwanted brain Hsp90 inhibition, which blocks opioid pain relief, by targeting spinal cord-specific Hsp90 isoforms. We found that Hsp90β and Grp94 alone regulate opioid signaling in the spinal cord, while Hsp90α regulates both brain and spinal cord opioid signaling. Thus, by delivering isoform-selective Hsp90β and Grp94 inhibitors by a systemic and translationally relevant route, we could enhance opioid pain relief by a similar 2–threefold while rescuing tolerance and not altering morphine-induced respiratory depression. By contrast, a systemic Hsp90α-selective inhibitor recapitulated brain inhibition, resulting in a loss of opioid anti-nociception. Together these findings establish the potential for Hsp90β and Grp94 inhibitors as opioid co-therapies, which would improve the therapeutic index of opioids, allowing for lower opioid doses with maintained analgesia and reduced side effects. Our model is summarized in Fig. 11.

There are a few caveats in interpreting these results. KUNG65 did show poor pharmacokinetic penetrance into the spinal cord at levels far lower than the K_D_ of this drug at Grp94. Since those K_D_ measurements were performed in purified protein, the drug may be more effective in a native context; alternatively, the system could be very sensitive, requiring low levels of occupancy to produce an effect. We did show that spinal knockdown of Grp94 abolished the benefit of systemic KUNG65, suggesting that the drug can engage the target effectively in the spinal cord (Fig. S3). It’s also possible that Hsp90 inhibition could alter morphine pharmacokinetics, although in that case, we would expect to see pain and side effects equally altered instead of some changed (pain, tolerance) and some not (reward).

Selectively boosting anti-nociceptive potency/efficacy in order to enable a dose-reduction strategy has precedent in the literature. Examples include demonstrated synergy between the cannabinoid receptor type 2 and opioid receptor for anti-nociception, without synergizing the side effects of either^19^. Further examples include selective inhibition of Gβγ signaling downstream of the mu opioid receptor, which similarly potentiates anti-nociception but not side effects^42^, morphine and clonidine synergy^43^, and using chemokine receptor antagonists to enable opioid dose-reduction^44^. These examples provide precedent and a reason to believe that dose-reduction with Hsp90 inhibitors would work. At the same time, to our knowledge, no such approaches are being used in the clinic, providing novelty and opportunity to use our findings.

Further factors argue for the clinical potential of our findings. Our earlier work suggests that other opioids, specifically oxymorphone, are regulated similarly by Hsp90 inhibition as morphine, albeit in different pain models and inhibitor route, while Hsp90 inhibition has no effect on gabapentin^15^. This finding suggests that a broad spectrum of opioid drugs could be improved. We’ve also shown that these inhibitors have no impact on opioid-induced respiratory depression, which is a key safety concern (Fig. 10). Our earlier work has shown that non-selective Hsp90 inhibitors given systemically mimic brain inhibition, resulting in a loss of opioid pain relief^16^; this means that our finding that isoform-selective inhibitors can be given systemically to recapitulate spinal inhibition is an important translational advance. Few patients are amenable to an intrathecal delivery route for chronic care, meaning that an isoform-selective approach is necessary to achieve the benefits of spinal Hsp90 inhibition.

One potential concern in adopting these isoform-selective inhibitors for therapy is the potential for on-target side effects. Indeed, early generation non-selective inhibitors like 17-AAG did not make it through clinical trials due to liver toxicity^45^. However, later generation compounds like CNF2024 were far better tolerated, suggesting this toxicity could be off-target rather than Hsp90-mediated^45^. In any case, isoform-selective inhibitors as we propose here should be far better tolerated than non-selective inhibitors, since each isoform has a distinct cellular location and pool of client proteins. This is further supported by a recent report that Hsp90α is the isoform responsible for the serious side effect of retinal degeneration associated with non-selective inhibitors^46^. Happily, our work suggests that Hsp90α is the very isoform that should be avoided for opioid therapy. Together these findings suggest that isoform-selective Hsp90 inhibitors could be feasible for chronic patient therapy. In addition, the rationale for reduced side effects above combined with literature from the cancer, inflammation, and neurodegeneration fields suggest these inhibitors could be improved options for the treatment of these diseases^47–49^.

These observations also raise the question of by what mechanism are these effects taking place. Our earlier work described an ERK-RSK kinase cascade that is not normally active in spinal cord, but becomes “unchained” with Hsp90 inhibition and promotes enhanced anti-nociception^16^. This cascade is presumably responsible for the wider enhancement in anti-nociceptive potency we see here across multiple acute and chronic pain models. Other possibilities could include alteration in opioid-induced prolactin secretion^50^ or suppression of TLR4 signaling^6^. At the same time, reward is primarily modulated by a ventral tegmental area-striatal circuit^17^ among other forebrain circuits, while constipation is primarily modulated by opioid receptors in the gut^18^; it thus makes sense why local spinal inhibition would not impact these regions and change the potency of those side effects. This leaves tolerance. Several studies have found that spinal circuits modulate opioid tolerance through various mechanisms, suggesting that local spinal inhibition could alter tolerance^51,52^. Spinal Hsp90 inhibition may favorably alter these mechanisms, blocking tolerance. Similarly, Hsp90 inhibition has been shown to be anti-inflammatory, and spinal neuroinflammation has been shown to contribute to opioid tolerance^7,53^. Alternately, enhanced anti-nociceptive efficacy could lead to less tolerance over time simply because the efficacy was higher to begin with; in support of this hypothesis, high efficacy opioid agonists have been shown to produce slower/less tolerance than low efficacy agonists^54^.

However, these hypotheses only address the slower tolerance seen over time with repeated treatment, they do not explain the tolerance rescue we observed. One potential clue for this rescue mechanism could be our earlier findings that both brain and spinal cord effects of Hsp90 inhibition on anti-nociception require rapid protein translation^14–16^. The translation inhibitor we used had no impact on anti-nociception in Vehicle-treated mice, suggesting that this mechanism is newly activated upon Hsp90 inhibitor treatment. The mechanisms we are uncovering could thus be parallel pathways to the normal/baseline anti-nociceptive signaling, which is further supported by our work on the spinal ERK-RSK cascade^16^. In the case of the tolerance rescue, we could be observing new pathways becoming turned on that have not developed tolerance as have the normal/baseline pathways. These new pathways could thus provide anti-nociceptive responsiveness even when other parts of the system remain tolerant. It’s also unclear at this point whether the tolerance reduction and the tolerance rescue share the same or different mechanisms. However, our observations in Fig. S1 suggest that these benefits to tolerance would not persist after discontinuing Hsp90 inhibitor treatment. Lastly, it is not at all clear why different Hsp90 isoforms are active in brain vs. spinal cord, whether inhibitor treatment alters isoform protein expression, and whether these isoforms differ in how they regulate opioid signaling. These questions will provide new basic science directions to follow while at the same time these findings can be used to inform a new clinical opioid dose-reduction approach.

### Supplementary Information

Supplementary Information.

## Data Availability

Data are provided within the manuscript or supplementary information file. Original raw data available upon request to the Corresponding Author.

## References

[1] Kroenke K, Krebs EE, Bair MJ (2009). Pharmacotherapy of chronic pain: A synthesis of recommendations from systematic reviews. Gen. Hosp. Psychiatry.

[2] Weiss RD, Rao V (2017). The prescription opioid addiction treatment study: What have we learned. Drug Alcohol Depend..

[3] Varga B, Streicher JM, Majumdar S (2021). Strategies towards safer opioid analgesics—A review of old and upcoming targets. Br. J. Pharmacol..

[4] Li J, Buchner J (2013). Structure, function and regulation of the hsp90 machinery. Biomed. J..

[5] Streicher JM (2019). The role of heat shock proteins in regulating receptor signal transduction. Mol. Pharmacol..

[6] Hutchinson MR (2009). Evidence for a role of heat shock protein-90 in toll like receptor 4 mediated pain enhancement in rats. Neuroscience.

[7] Lewis SS (2010). Evidence that intrathecal morphine-3-glucuronide may cause pain enhancement via toll-like receptor 4/MD-2 and interleukin-1beta. Neuroscience.

[8] Grace PM (2017). Protraction of neuropathic pain by morphine is mediated by spinal damage associated molecular patterns (DAMPs) in male rats. Brain Behav. Immun..

[9] Abul-Husn NS (2011). Chronic morphine alters the presynaptic protein profile: Identification of novel molecular targets using proteomics and network analysis. PloS One.

[10] Koshimizu TA (2010). Inhibition of heat shock protein 90 attenuates adenylate cyclase sensitization after chronic morphine treatment. Biochem. Biophys. Res. Commun..

[11] Zhang Y (2020). Hsp90beta positively regulates mu-opioid receptor function. Life Sci..

[12] Streicher JM (2019). The role of heat shock protein 90 in regulating pain, opioid signaling, and opioid antinociception. Vitam. Horm..

[13] Lei W (2017). Heat shock protein 90 (Hsp90) promotes opioid-induced anti-nociception by an ERK mitogen activated protein kinase (MAPK) mechanism in mouse brain. J. Biol. Chem..

[14] Lei W (2019). The alpha isoform of heat shock protein 90 and the Co-chaperones p23 and Cdc37 Promote Opioid Anti-nociception in the Brain. Front. Mol. Neurosci..

[15] Stine C (2020). Heat shock protein 90 inhibitors block the anti-nociceptive effects of opioids in mouse chemotherapy-induced neuropathy and cancer bone pain models. Pain.

[16] Duron DI (2020). Inhibition of Hsp90 in the spinal cord enhances the antinociceptive effects of morphine by activating an ERK-RSK pathway. Sci. Signal..

[17] Sandweiss AJ (2017). Genetic and pharmacological antagonism of NK1 receptor prevents opiate abuse potential. Mol. Psychiatry.

[18] Streicher JM, Bilsky EJ (2017). Peripherally acting micro-opioid receptor antagonists for the treatment of opioid-related side effects: Mechanism of action and clinical implications. J. Pharm. Pract..

[19] Grenald SA (2017). Synergistic attenuation of chronic pain using mu opioid and cannabinoid receptor 2 agonists. Neuropharmacology.

[20] Burlison JA, Neckers L, Smith AB, Maxwell A, Blagg BS (2006). Novobiocin: Redesigning a DNA gyrase inhibitor for selective inhibition of hsp90. J. Am. Chem. Soc..

[21] Mishra SJ (2021). Selective inhibition of the Hsp90alpha isoform. Angew Chem. Int. Ed. Engl..

[22] Mishra SJ (2021). The development of Hsp90beta-selective inhibitors to overcome detriments associated with pan-Hsp90 inhibition. J. Med. Chem..

[23] Duerfeldt AS (2012). Development of a Grp94 inhibitor. J. Am. Chem. Soc..

[24] Ananthan S (2012). 14-Alkoxy- and 14-acyloxypyridomorphinans: Mu agonist/delta antagonist opioid analgesics with diminished tolerance and dependence side effects. J. Med. Chem..

[25] Lei W (2017). Heat-shock protein 90 (Hsp90) promotes opioid-induced anti-nociception by an ERK mitogen-activated protein kinase (MAPK) mechanism in mouse brain. J. Biol. Chem..

[26] Duron DI, Hanak F, Streicher JM (2020). Daily intermittent fasting in mice enhances morphine-induced anti-nociception while mitigating reward, tolerance, and constipation. Pain.

[27] Chaplan SR, Bach FW, Pogrel JW, Chung JM, Yaksh TL (1994). Quantitative assessment of tactile allodynia in the rat paw. J. Neurosci. Methods.

[28] Lei W, Vekariya RH, Ananthan S, Streicher JM (2019). A novel mu-delta opioid agonist demonstrates enhanced efficacy with reduced tolerance and dependence in mouse neuropathic pain models. J. Pain.

[29] Hylden JL, Wilcox GL (1980). Intrathecal morphine in mice: A new technique. Eur. J. Pharmacol..

[30] Wilson DM, Wang X, Walsh E, Rourick RA (2001). High throughput log D determination using liquid chromatography-mass spectrometry. Comb. Chem. High Throughput Screen..

[31] Zhou L, Yang L, Tilton S, Wang J (2007). Development of a high throughput equilibrium solubility assay using miniaturized shake-flask method in early drug discovery. J. Pharm. Sci..

[32] Di L, Kerns EH, Li SQ, Petusky SL (2006). High throughput microsomal stability assay for insoluble compounds. Int. J. Pharm..

[33] Lei W, Vekariya RH, Ananthan S, Streicher JM (2020). A novel mu-delta opioid agonist demonstrates enhanced efficacy with reduced tolerance and dependence in mouse neuropathic pain models. J. Pain.

[34] Stefanucci A (2020). Potent, efficacious, and stable cyclic opioid peptides with long lasting antinociceptive effect after peripheral administration. J. Med. Chem..

[35] Vekariya RH (2020). Synthesis and structure-activity relationships of 5'-Aryl-14-alkoxypyridomorphinans: Identification of a mu opioid receptor agonist/delta opioid receptor antagonist ligand with systemic antinociceptive activity and diminished opioid side effects. J. Med. Chem..

[36] LaVigne JE, Hecksel R, Keresztes A, Streicher JM (2021). Cannabis sativa terpenes are cannabimimetic and selectively enhance cannabinoid activity. Sci. Rep..

[37] Tanguturi P (2021). Discovery of novel delta opioid receptor (DOR) inverse agonist and irreversible (non-competitive) antagonists. Molecules (Basel, Switzerland).

[38] Berge OG (2011). Predictive validity of behavioural animal models for chronic pain. Br. J. Pharmacol..

[39] Yuan SB (2014). Gp120 in the pathogenesis of human immunodeficiency virus-associated pain. Ann. Neurol..

[40] Warner M, Trinidad JP, Bastian BA, Minino AM, Hedegaard H (2016). Drugs most frequently involved in drug overdose deaths: United States, 2010–2014. Natl. Vital Stat. Rep..

[41] Crowley VM, Huard DJE, Lieberman RL, Blagg BSJ (2017). Second generation Grp94-selective inhibitors provide opportunities for the inhibition of metastatic cancer. Chemistry.

[42] Hoot MR (2013). Inhibition of Gbetagamma-subunit signaling potentiates morphine-induced antinociception but not respiratory depression, constipation, locomotion, and reward. Behav. Pharmacol..

[43] Stone LS, German JP, Kitto KF, Fairbanks CA, Wilcox GL (2014). Morphine and clonidine combination therapy improves therapeutic window in mice: Synergy in antinociceptive but not in sedative or cardiovascular effects. PloS One.

[44] Eisenstein TK (2020). Chemokine receptor antagonists in combination with morphine as a novel strategy for opioid dose reduction in pain management. Mil. Med..

[45] Sidera K, Patsavoudi E (2014). HSP90 inhibitors: Current development and potential in cancer therapy. Recent Pat Anticancer Drug Discov..

[46] Wu Y (2020). The molecular chaperone Hsp90alpha deficiency causes retinal degeneration by disrupting Golgi organization and vesicle transportation in photoreceptors. J. Mol. Cell Biol..

[47] Mishra, S. J. *et al.* Hsp90beta-selective inhibitors exhibit nanomolar potency and overcome detriments associated with pan-Hsp90 inhibition. *J. Med. Chem.* (2019). 10.1021/acs.jmedchem.0c01700 PMC8996186 33428418

[48] Campanella C (2018). Heat shock proteins in Alzheimer's disease: Role and targeting. Int. J. Mol. Sci..

[49] Mu H, Wang L, Zhao L (2016). HSP90 inhibition suppresses inflammatory response and reduces carotid atherosclerotic plaque formation in ApoE mice. Cardiovasc. Ther..

[50] Chen Y (2020). The prolactin receptor long isoform regulates nociceptor sensitization and opioid-induced hyperalgesia selectively in females. Sci. Transl. Med..

[51] Xu JT (2014). Opioid receptor-triggered spinal mTORC1 activation contributes to morphine tolerance and hyperalgesia. J. Clin. Investig..

[52] Zhai ML, Chen Y, Liu C, Wang JB, Yu YH (2018). Spinal glucocorticoid receptorregulated chronic morphine tolerance may be through extracellular signalregulated kinase 1/2. Mol. Med. Rep..

[53] Pan Y (2016). Metformin reduces morphine tolerance by inhibiting microglial-mediated neuroinflammation. J. Neuroinflammation..

[54] Pawar M (2007). Opioid agonist efficacy predicts the magnitude of tolerance and the regulation of mu-opioid receptors and dynamin-2. Eur. J. Pharmacol..

