# Supplementary material for: Inhibiting spinal cord-specific hsp90 isoforms reveals a novel strategy to improve the therapeutic index of opioid treatment
Source: Sci Rep. 2024 Jun 26;14:14715. doi: 10.1038/s41598-024-65637-6 (PMC11208559; doi:10.1038/s41598-024-65637-6)
Supplement: Supplementary file 1 — Supplementary Information. [file 41598_2024_65637_MOESM1_ESM.pdf]

Supplementary Information for:

Inhibiting Spinal Cord-Specific Hsp90 Isoforms Reveals a Novel Strategy to Improve the Therapeutic Index of Opioid Treatment

David I. Duron[1#], Parthasaradhireddy Tanguturi[1#], Christopher S. Campbell[1#], Kerry Chou[1#], Paul Bejarano[1], Katherin A. Gabriel[1], Jessica L. Bowden[1], Sanket Mishra[2], Christopher Brackett[2], Deborah Barlow[3], Karen L. Houseknecht[3], Brian S.J. Blagg[2], and John M. Streicher[1,4,*]

[1]Department of Pharmacology, College of Medicine, University of Arizona, Tucson AZ USA
[2]Department of Chemistry and Biochemistry, College of Science, University of Notre Dame, Notre Dame IN USA
[3]Department of Biomedical Sciences, College of Osteopathic Medicine, University of New England, Biddeford ME USA
[4]Comprehensive Center for Pain and Addiction, University of Arizona, Tucson AZ USA
[#]These authors contributed equally to the work

***Corresponding Author:** John M. Streicher, Ph.D.; University of Arizona College of Medicine, Box 245050, LSN563, 1501 N. Campbell Ave., Tucson AZ 85724. Ph: (520)-626-7495. Email: jstreicher@arizona.edu

ORCID: John Streicher 0000-0002-4173-7362

Keywords: Heat shock protein 90, opioid, anti-nociception, reward, constipation, tolerance, therapeutic index, isoforms

Running Title: Inhibiting Spinal Hsp90 Isoforms Improves Opioid Therapy

Number of Figures: 11 main, 8 supplemental

Figure S1

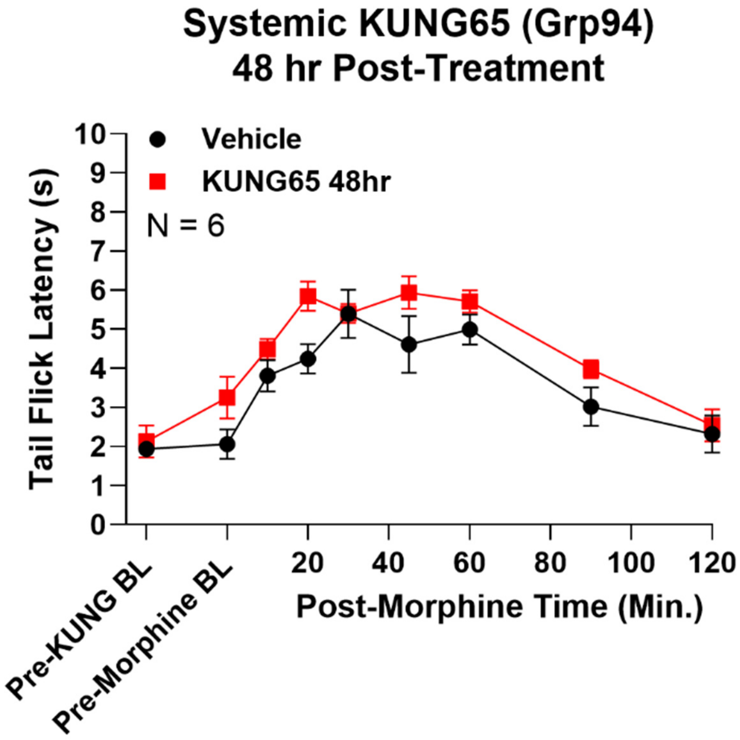

Figure S1: Enhanced morphine anti-nociception after Grp94 inhibitor treatment dissipates by 48 hours post-treatment. Male and female CD-1 mice were injected with 1 mg/kg KUNG65 or Vehicle control i.v., followed by 48 hrs of treatment, then 3.2 mg/kg morphine s.c. and a tail flick timecourse. Males and females did not differ by 2 Way ANOVA ($p > 0.05$), so all male and female data were combined. The data were displayed as the mean ± SEM with the sample size of mice/group noted in the graph; the experiment was completed with 1 technical replicate. The anti-nociception after 48 hrs of KUNG65 treatment was not significantly different from control ($p > 0.05$), suggesting that the benefits of the treatment wear off between 24 and 48 hrs post-treatment.

Figure S2

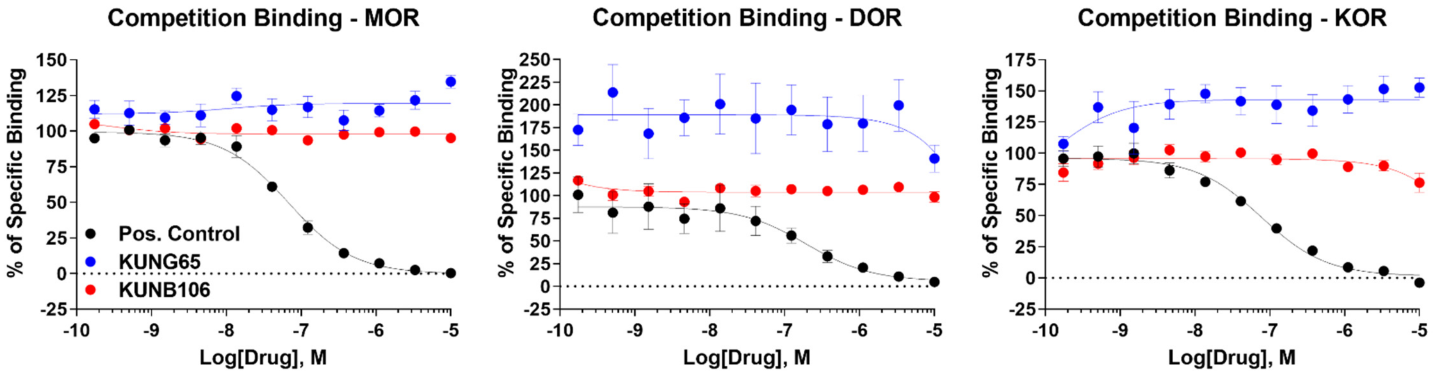

Figure S2: The Grp94 and Hsp90β inhibitors KUNG65 and KUNB106 do not bind to the opioid receptors. KUNG65, KUNB106, and positive control (naloxone for mu opioid receptor [MOR] and delta opioid receptor [DOR], U50,488 for kappa opioid receptor [KOR]) were competed against ^{3}H-diprenorphine at all 3 human opioid receptors using competition radioligand binding (see Methods). The data were shown as the mean ± SEM of N = 3 independent experiments. Positive control compounds displayed competition as expected, validating the assay (K_I values: MOR = 34 ± 3 nM; DOR = 120 ± 44 nM; KOR = 37 ± 4 nM). KUNG65 and KUNB106 did not display competition up to a 10 μM concentration, ruling out opioid receptor binding as a potential confound for our findings.

Figure S3

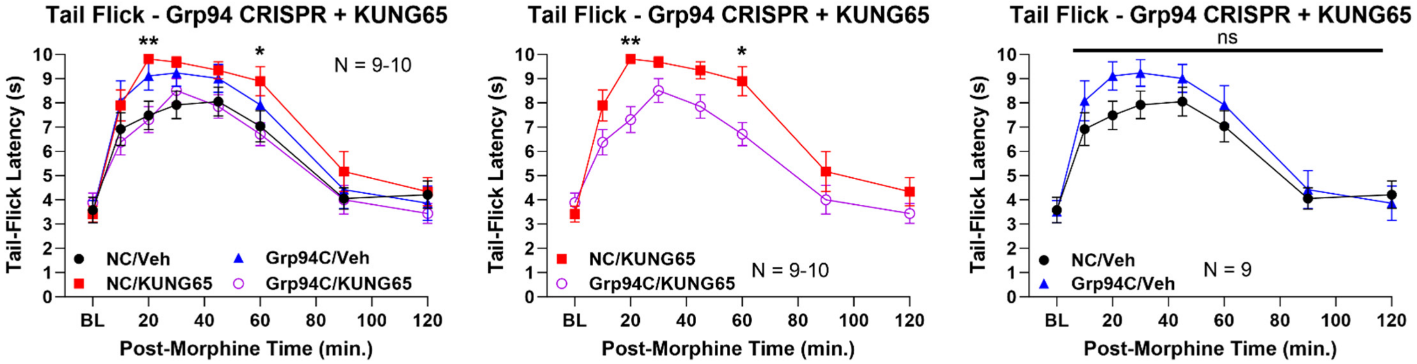

Figure S3: Spinal Grp94 knockdown blocks the effect of systemic KUNG65 treatment. Male and female CD-1 mice were injected i.t. with CRISPR constructs targeting the Grp94 isoform or Negative Control (NC) as described in the Methods. On day 9, the mice were injected with Vehicle or 1 mg/kg KUNG65 i.v., 24 hrs, then 3.2 mg/kg morphine s.c. and a tail flick timecourse. Males and females did not differ by 2 Way ANOVA ($p > 0.05$), so all male and female data were combined. The data were displayed as the mean ± SEM with the sample size of mice/group noted in the graph; the experiment was completed with 2 technical replicates. *, ** = $p < 0.05$, 0.01 vs. same time point Grp94C/KUNG65 group by RM 2-Way ANOVA with Dunnett's *post hoc* test. *Left*: all groups shown; *Center:* only KUNG65 groups shown; *Right:* only Vehicle groups shown. Grp94 knockdown in the spinal cord completely blocked the enhancement in morphine pain relief, with the Grp94C/KUNG65 group returned to the same level as the NC/Veh group.

Figure S4

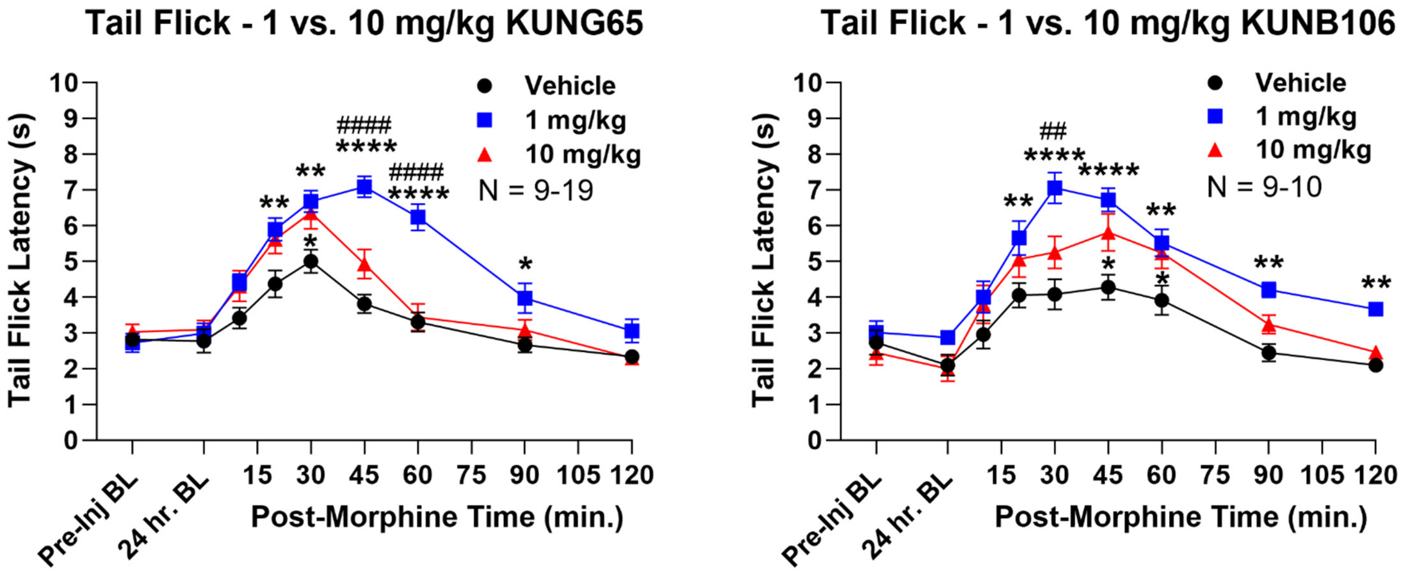

Figure S4: Higher dose Hsp90 inhibitor treatment provides less benefit than a 1 mg/kg dose. Male and female CD-1 mice were injected with Vehicle control, 1 or 10 mg/kg KUNG65 (*left*), or 1 or 10 mg/kg KUNB106 (*right*) i.v., 24 hrs, followed by 3.2 mg/kg morphine s.c. and a tail flick timecourse. Males and females did not differ by 2 Way ANOVA ($p > 0.05$), so all male and female data were combined. The data were displayed as the mean ± SEM with the sample size of mice/group noted in the graph; the experiment was completed with 2-4 technical replicates. *, **, **** = $p < 0.05$, 0.01, 0.0001 vs. same time point Vehicle group; ##, #### = $p < 0.01$, 0.0001 vs. same time point 1 mg/kg group; both by RM 2 Way ANOVA with Dunnett's *post hoc* test. For both inhibitors, the 10 mg/kg dose is significantly elevated over Vehicle, but the 1 mg/kg group is significantly increased over both Vehicle and the 10 mg/kg group. This observation suggests that 1 mg/kg is near the top of the dose curve, and higher doses provide less benefit in a U-shaped dose-response.

Figure S5

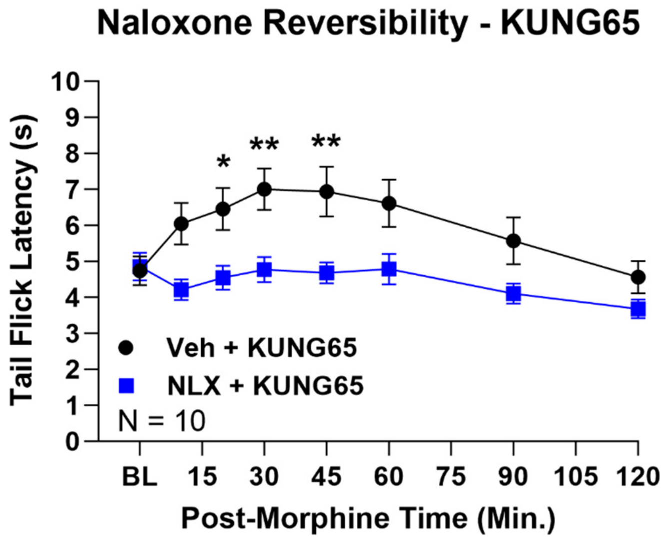

Figure S5: Enhanced morphine pain relief with Hsp90 inhibitor treatment is naloxone-reversible. Male and female CD-1 mice were all injected with either 1 mg/kg KUNG65 (*left*) or 1 mg/kg KUNB106 (*right*) i.v., 24 hrs, then with either Vehicle or 2 mg/kg naloxone i.p. for 10 min. Following this 10 min treatment, all mice were injected with 3.2 mg/kg morphine s.c. and a tail flick timecourse performed. Males and females did not differ by 2 Way ANOVA ($p > 0.05$), so all male and female data were combined. The data were displayed as the mean ± SEM with the sample size of mice/group noted in the graph; the experiment was completed with 2 technical replicates. *, **, **** = $p < 0.05$, 0.01, 0.0001 vs. same time point Naloxone group by RM 2 Way ANOVA with Sidak's *post hoc* test. Naloxone treatment fully reversed all anti-nociception back to baseline (BL) levels, demonstrating that all pain relief is achieved via the opioid system in these experiments.

Figure S6

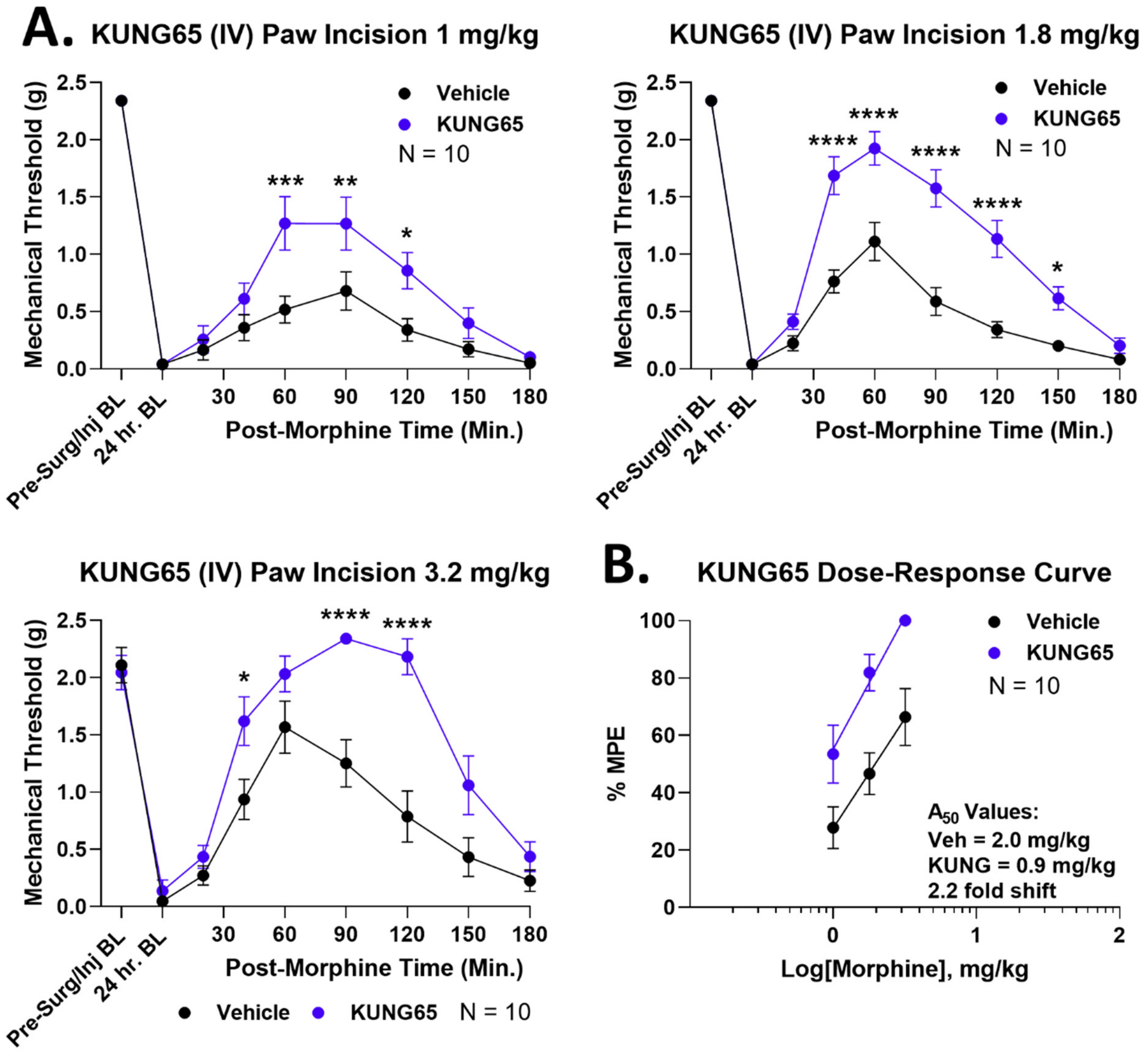

Figure S6: Systemic Grp94 inhibition enhances morphine anti-nociception in paw incision pain. Male and female CD-1 mice were treated with paw incision surgery and 1 mg/kg KUNG65 or Vehicle control i.v., 24 hrs, followed by 1 – 3.2 mg/kg s.c. morphine and a von Frey mechanical allodynia timecourse performed. Males and females did not differ by 2 Way ANOVA ($p > 0.05$), so all male and female data were combined. The data were presented as the mean ± SEM, with sample sizes of mice/group noted in each graph; experiments performed in 2 technical replicates per dose. **A)** Individual dose curves shown (no transformation). *, **, ***, **** = $p < 0.05$, 0.01, 0.001, 0.0001 vs. same time point Vehicle group by 2 Way RM ANOVA with Sidak's *post hoc* test. KUNG65 treatment consistently elevated anti-nociception. **B)** The data were transformed into peak %MPE and used to construct dose/response curves, with linear regression for A_{50} calculation (see Methods). A_{50}: Vehicle = 2.0 (1.4 – 3.0) mg/kg, KUNG65 = 0.9 (0.5 – 1.2) mg/kg; 2.2-fold improvement in morphine potency.

Figure S7

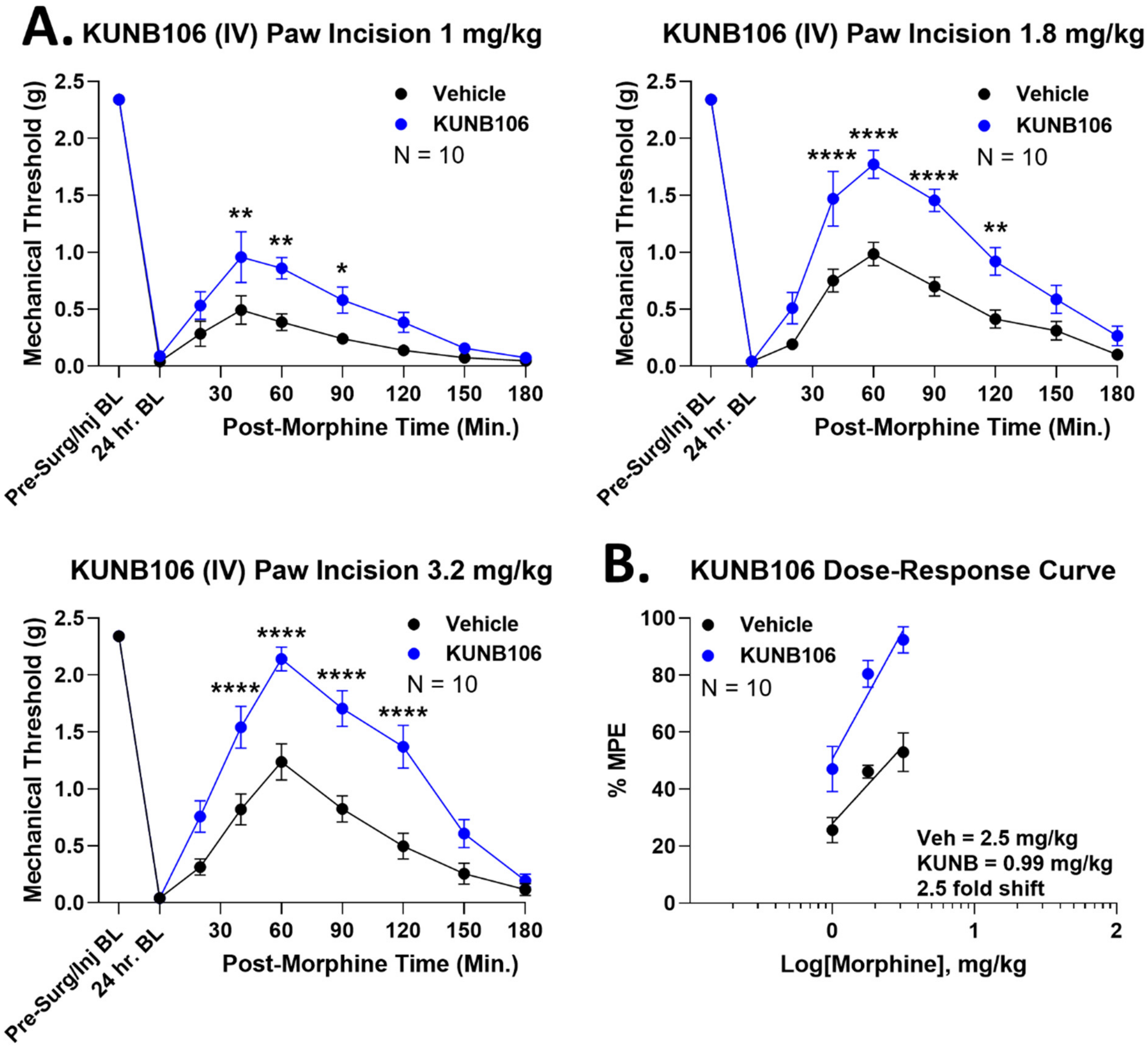

Figure S7: Systemic Hsp90β inhibition enhances morphine anti-nociception in paw incision pain. Male and female CD-1 mice were treated with paw incision surgery and 1 mg/kg KUNB106 or Vehicle control i.v., 24 hrs, followed by 1 – 3.2 mg/kg s.c. morphine and a von Frey mechanical allodynia timecourse performed. Males and females did not differ by 2 Way ANOVA ($p > 0.05$), so all male and female data were combined. The data were presented as the mean ± SEM, with sample sizes of mice/group noted in each graph; experiments performed in 2 technical replicates per dose. **A)** Individual dose curves shown (no transformation). *, **, **** = $p < 0.05$, 0.01, 0.0001 vs. same time point Vehicle group by 2 Way RM ANOVA with Sidak's *post hoc* test. KUNB106 treatment consistently elevated anti-nociception. **B)** The data were transformed into peak %MPE and used to construct dose/response curves, with linear regression for A_{50} calculation (see Methods). A_{50}: Vehicle = 2.5 (2.0 – ∞) mg/kg, KUNB106 = 0.99 (∞ – ∞) mg/kg; 2.5 fold improvement in morphine potency.

Figure S8

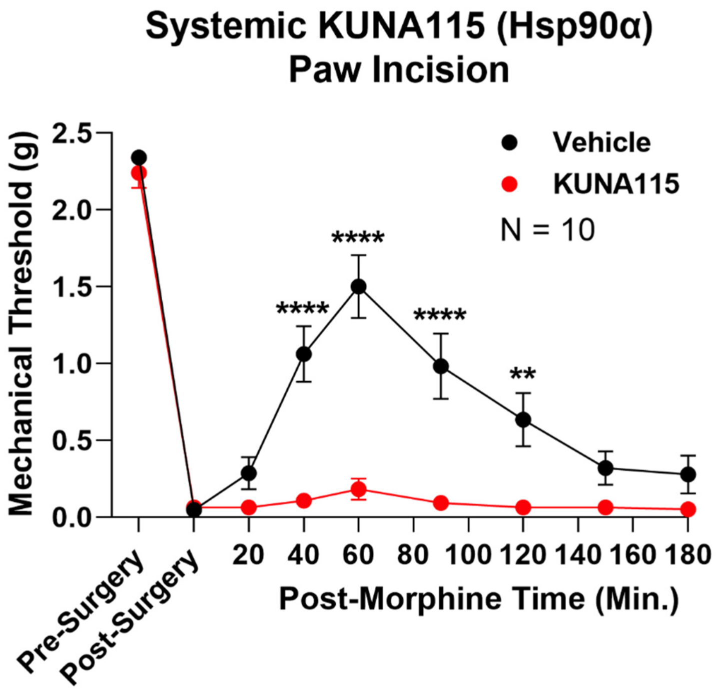

Figure S8: Systemic Hsp90α inhibition blocks opioid anti-nociception. Male and female CD-1 mice had paw incision performed, then were injected with 1 mg/kg KUNA115 or Vehicle i.v., 24 hrs, followed by 3.2 mg/kg morphine s.c. and a von Frey mechanical allodynia timecourse performed. Males and females did not differ by 2 Way ANOVA ($p > 0.05$), so all male and female data were combined. The data were presented as the mean ± SEM with the sample size of mice/group noted in the graph (performed in 2 technical replicates). **, **** = $p < 0.01, 0.0001$ vs. same time point Vehicle group by 2 Way RM ANOVA with Sidak's *post hoc* test. Unlike Hsp90β and Grp94 inhibition, systemic Hsp90α inhibition via KUNA115 completely blocked opioid anti-nociception in this model.
